# Evaluation and Validation of the Prognostic Value of Serum Albumin to Globulin Ratio in Patients With Cancer Cachexia: Results From a Large Multicenter Collaboration

**DOI:** 10.3389/fonc.2021.707705

**Published:** 2021-09-10

**Authors:** Hai-Lun Xie, Qi Zhang, Guo-Tian Ruan, Yi-Zhong Ge, Chun-Lei Hu, Meng-Meng Song, Chun-Hua Song, Xi Zhang, Xiao-Wei Zhang, Xiang-Rui Li, Kang-Ping Zhang, Tong Liu, Ming Yang, Meng Tang, Hong-Xia Xu, Han-Ping Shi

**Affiliations:** ^1^Department of Gastrointestinal Surgery/Department of Clinical Nutrition, Beijing Shijitan Hospital, Capital Medical University, Beijing, China; ^2^Beijing International Science and Technology Cooperation Base for Cancer Metabolism and Nutrition, Beijing Shijitan Hospital, Capital Medical University, Beijing, China; ^3^Department of Clinical Nutrition, Daping Hospital, Army Medical University, Chongqing, China; ^4^Department of Epidemiology, College of Public Health, Zhengzhou University, Zhenzhou, China

**Keywords:** albumin–globulin ratio, prognostic, cachexia, inflammation, nutrition, cancer

## Abstract

**Background:**

Recently, albumin–globulin ratio (AGR), a serological indicator that reflects nutritional status and systemic inflammatory, has been reported to be associated with the prognosis of various cancers. However, there is currently no research report on its relationship with cancer cachexia.

**Objectives:**

This study aimed to explore the prognostic value of AGR in patients with cancer cachexia through a multicenter retrospective analysis.

**Methods:**

We recruited 2,364 patients with cancer cachexia and randomly divided the patients into training and validation cohorts at a ratio of 7:3. The optimal stratification method was used to determine the optimal cutoff value of AGR. The survival curve was evaluated by the Kaplan–Meier method. Cox regression proportional-hazards model was used to determine independent prognostic factors in patients with cancer cachexia. The time-dependent receiver operating characteristic curve was used to compare the prognostic performance of different malnutrition evaluation tools.

**Results:**

The optimal cutoff value of AGR is 1.24 in patients with cancer cachexia. Increasing AGR was associated with survival in a dose–response manner with a forward L-shape. Compared with the high AGR group, the low AGR group had a shorter overall survival; and there was consistency in training and validation cohorts. In the stratified analysis of TNM stage, AGR has good prognostic distinguishing ability for advanced patients. Multivariate survival analysis determined that low AGR was an independent risk factor affecting all-cause mortality in patients with cancer cachexia. In addition, compared with other malnutrition evaluation tools, AGR could effectively stratify the prognosis of patients with cancer cachexia.

**Conclusion:**

AGR was an independent prognostic factor affecting patients with cancer cachexia, especially in advanced patients. Compared with other malnutrition evaluation tools, AGR can effectively stratify the prognosis of patients with cancer cachexia.

## Introduction

According to the World Health Organization (1), cancer is the second leading cause of death globally and is responsible for about 10 million deaths per year. Globally, about one in six deaths is due to cancer. In the past year, there were an estimated 19.3 million new cancer cases and nearly 10 million cancer deaths worldwide (2). In China, cancer is still one of the major killers affecting national health. There are about 3.929 million new cancer cases and 2.338 million cancer deaths every year, and the number is still growing (3). Despite the development of cancer treatment technologies such as surgery, chemoradiotherapy, and molecular targeting, the survival of patients with cancer is still unsatisfactory.

Cancer cachexia, a destructive metabolic syndrome of cancer, is one of the main causes of unsatisfactory outcomes in patients with cancer. It has been reported that 50%~80% of patients with cancer experience varying degrees of cancer cachexia, with an even higher proportion in patients with advanced cancer (4). Patients suffering from cancer cachexia are at increased risk of death, especially patients with refractory cachexia. Argiles et al. (5) estimated that deaths caused by cancer cachexia accounted for approximately 20% of all cancer deaths. In addition, cancer cachexia also limits the treatment options because it can enhance the toxic side effects of radiotherapy-, chemotherapy-, and molecular-targeted therapies. What makes people more entangled is that chemotherapy can also induce cancer cachexia, leading to a more serious vicious circle (6, 7). Although it is widely accepted that cancer cachexia increases mortality in patients, cancer cachexia has not yet been actively treated. There is still a lack of effective markers to predict the prognosis of cancer cachexia and to help select the best intervention strategies, especially convenient, accessible, and inexpensive serum markers.

In recent years, albumin–globulin ratio (AGR), a serological indicator that reflects nutritional status and systemic inflammatory, has been reported to be associated with the prognosis of various cancers (8, 9). Because of its simplicity, cheapness, and easy availability, AGR has attracted increasing attention. Serum albumin (ALB) is not only the most common nutritional indicator; some studies have also found that ALB was associated with the activation of systemic inflammation during tumor proliferation and invasion (10–12). In addition, hypoproteinemia has been reported to be associated with poor prognosis of various cancers (13, 14). Serum globulin (GLB), a proinflammatory protein that contains a variety of inflammatory mediators, such as chemokines, cytokines, and other small inflammatory proteins, plays a vital role in immunity and inflammation (15, 16). Studies have shown that local and systemic immune responses in cancer-related inflammation are associated with an increase in these inflammatory mediators (17, 18). Therefore, AGR, which integrates nutritional and inflammatory status, is a potential indicator for predicting the prognosis of patients with cancer cachexia. So far, no studies have reported the relationship between AGR and patients with cancer cachexia. Therefore, we first conducted the large-scale multicenter retrospective study to evaluate and validate the prognostic value of AGR in patients with cancer cachexia.

## Material and Methods

### Study Population

The study included pathologically confirmed patients with cancer from more than 40 clinical centers in China between June 2012 and December 2019. In this study, we screened patients strictly according to the exclusion and inclusion criteria. Inclusion criteria included the following: 1) patients who met the criteria for diagnosis of cancer cachexia; 2) patients with complete and available clinicopathological parameters; and 3) patients with complete follow-up data. Exclusion criteria included the following: 1) patients younger than 18 years; 2) patients with obvious clinical evidence of infection or inflammation; 3) patients who underwent organ transplantation; 4) pregnant women; and 5) patients admitted to the intensive care unit (ICU) at the beginning of recruitment. The study was approved by the institutional review boards of all participating institutions, and all patients provided written informed consent.

### Data Collection

We collected complete clinicopathological parameters by consulting patients’ medical records. Basic information included gender, age, height, weight, body mass index (BMI), and family history of cancer. Comorbidities included hypertension and diabetes. Life habits included smoking and drinking. Cancer characteristics included cancer type and tumor–node–metastasis (TNM) stage system. Malnutrition evaluation tools included AGR, patient-generated subjective global assessment (PG-SGA), handgrip strength (HGS), Karnofsky Performance Status (KPS), and mid-arm circumference (MAC). Other prognostic evaluation tools included neutrophil–lymphocyte ratio (NLR) and platelet–lymphocyte ratio (PLR). These are all collected in the research baseline. Laboratory serological tests included neutrophil count, lymphocyte count, platelet count, white blood cells (WBC), serum total protein levels (TB), ALB, and GLB. All laboratory serological tests were collected and tested within 1 week prior to treatment. Treatment methods included surgery, radiotherapy, and chemotherapy, which were collected during the follow-up. The TNM stage system was determined according to the 8th edition staging criteria of the American Joint Committee on Cancer (AJCC). The AGR was defined as serum albumin levels (g/L)/serum globulin levels (g/L). The NLR was defined as neutrophil count (10^9^/L)/lymphocyte count (10^9^/L). The PLR was defined as platelet count (10^9^/L)/lymphocyte count (10^9^/L).

### Cachexia Definition and Assessment

In 2011, Fearon et al. (19) proposed the definition and diagnostic criteria of cancer cachexia in the International Consensus on Cachexia, which has since been widely recognized. The cachexia is defined as a multifactorial syndrome characterized by persistent skeletal muscle loss (with or without adipose tissue loss) that cannot be completely relieved by conventional nutritional support and gradually leads to functional impairment. The diagnosis of cancer cachexia mainly revolves around three aspects: weight loss, BMI decline, and sarcopenia. The specific contents are as follows: a) weight loss >5% over past 6 months (eliminate simple starvation); b) BMI <20 and any degree of weight loss >2%; and c) appendicular skeletal muscle index diagnosis of sarcopenia (males <7.26 kg/m^2^; females <5.45 kg/m^2^) and any degree of weight loss >2%. As long as one of the above is present, a patient can be diagnosed with cachexia. In this study, patients with cancer cachexia were screened strictly according to the criteria. In our study, the appendicular skeletal muscle index was calculated based on previous studies (20, 21).

### Follow-Up

In this study, all patients were followed up regularly by telephone or outpatient visits. Telephone follow-up mainly included inquiries about survival and treatment, and outpatient follow-up mainly included physical examination, blood test, and imaging examination, as well as endoscopic examination when necessary. The deadline for follow-up of this study was September 30, 2019. The main outcome of this study was overall survival (OS), which was defined as the time interval between first diagnosis and death or last follow-up.

### Statistical Analysis

Descriptive data conforming to a normal distribution are presented as a mean [± standard deviation (SD)], otherwise as a median (interquartile interval) or frequency (percentage). Category variables were analyzed using either chi-square test or Fisher’s exact test, and continuous variables were analyzed using Student’s t-test. Restricted cubic spine function with 3 knots was performed to evaluate the effects for continuous AGR on survival. Based on the survival status, the optimal stratification method was used to determine the optimal cutoff value of AGR. The survival curve was evaluated by the Kaplan–Meier method, and the survival difference between the groups was evaluated by the log-rank test. Univariate and multivariate Cox regression proportional-hazards models were used to determine independent prognostic factors in patients with cancer cachexia. In addition, stratified analysis was used to evaluate the relationship between AGR and all-cause mortality in different subgroups, and the interaction was used to investigate whether there was an association between AGR and different clinical parameters. We also use the quartile method and continuous variable method to test the trend, and use the Wald test to evaluate the statistical significance. The time-dependent receiver operating characteristic (ROC) curve was used to compare the prognostic performance of different malnutrition evaluation tools. A *p* < 0.05 for both sides was considered statistically significant. All statistical analyses were performed using SPSS version 24.0 (IBM Corporation, Armonk, NY, USA) and R version 3.5.3 (https://www.r-project.org/).

## Results

### Distribution of Albumin–Globulin Ratio in Patients With Cancer Cachexia

**Figure 1:**
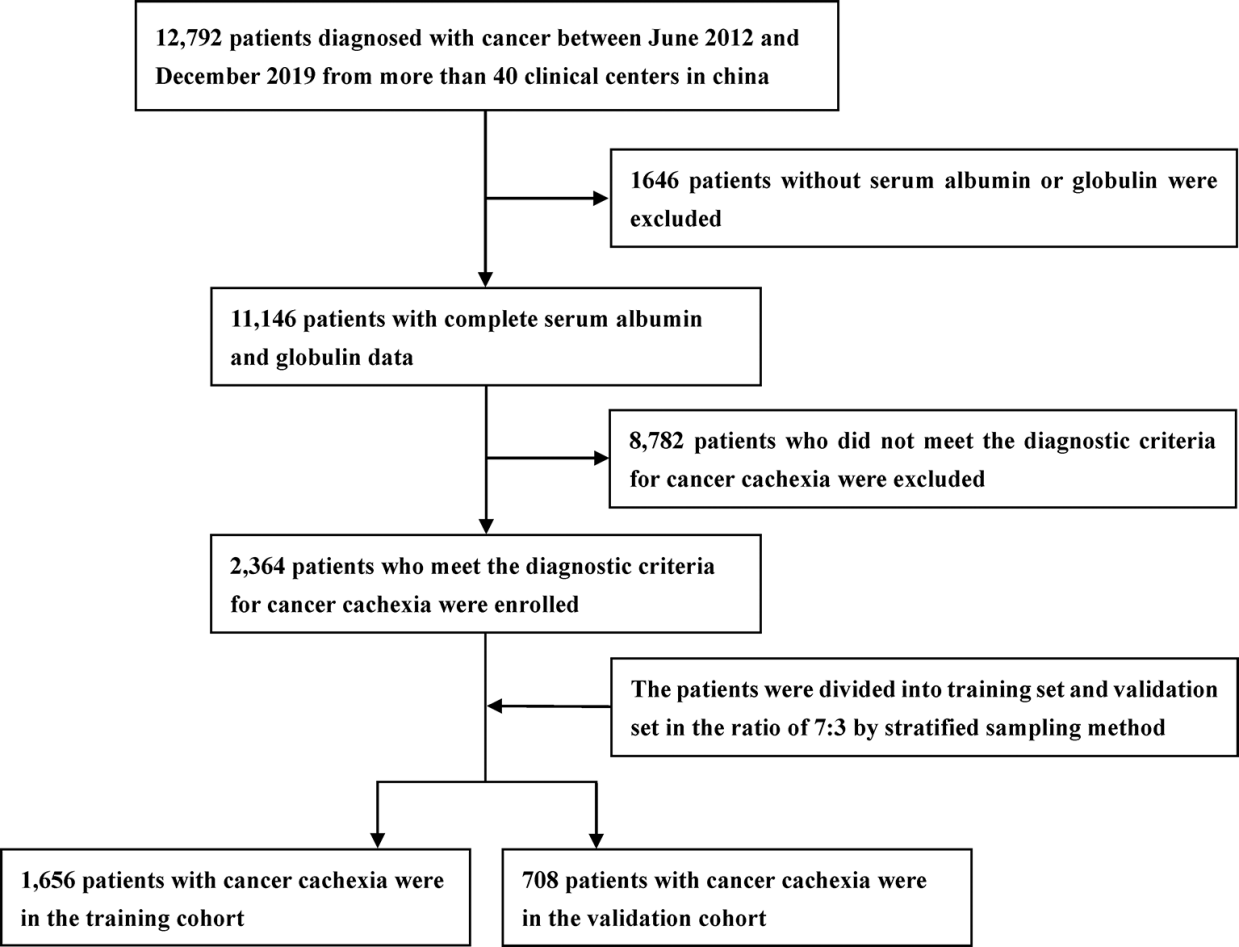
Study design.

**Figure 2:**
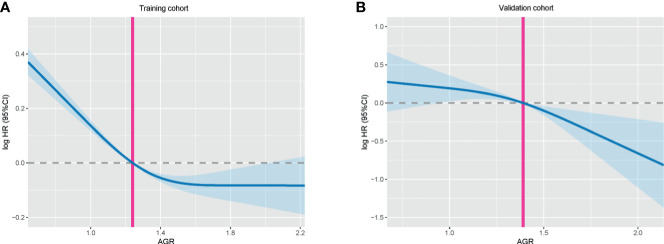
The association between AGR (continuous) and hazard risk of overall survival in training cohort **(A)** and validation cohort **(B)**. The splines were adjusted by gender, age, BMI, TNM stage, surgery, radiotherapy, chemotherapy, family history, hypertension, diabetes, smoke, and alcohol. AGR, albumin–globulin ratio; BMI, body mass index.

A total of 2,364 patients with cancer cachexia were enrolled. We divided these populations into a training cohort (1,656 cases) and a validation cohort (708 cases) using random sampling in a 7:3 ratio (**Figure 1**). **Table 1** shows the clinicopathological characteristics of the training and validation populations. Most of the clinicopathological characteristics were in good agreement between the two cohorts. We compared the AGR level of patients with and without cachexia among different cancer types, and the results showed that the AGR levels were generally low in patients with cachexia, especially in gastrointestinal cancers (**Supplementary Figure 1A**). In addition, we explored the distribution of AGR in patients with cancer cachexia through the restricted cubic spine function. When AGR was analyzed as a continuous variable, there was a dose–response relationship between AGR and survival with a positive L-type (**Figures 2A, B**). With the increase of AGR, the OS of patients gradually decreased. There was an obvious inflection point between 1.2 and 1.4, and the survival rate of the patients before this was significantly lower than that of the patients after. In addition, we further determined the optimal cutoff value of AGR as 1.24 by using the optimal stratification method based on the survival status, which was consistent with the results of the restricted cubic spline plots (**Supplementary Figure 2**).

**Table 1:** Demographics and clinicopathological characteristics of cachexia patients.

Characteristic	Case n = 2,364	Training cohort n = 1,656	Validation cohort n = 708	*p*
Population characteristic				
Gender, male, n (%)	1,379 (58.3%)	973 (58.8%)	406 (57.3%)	0.524
Age, years, mean (SD)	58.41 (12.15)	58.63 (11.79)	57.88 (12.94)	0.168
BMI, kg/m^2^, mean (SD)	20.88 (3.26)	20.88 (3.25)	20.88 (3.29)	0.993
SMI, kg/m^2^, median (IQR)	6.65 (1.56)	6.66 (1.6)	6.61 (1.49)	0.640
Family history, yes, n (%)	350 (14.8%)	243 (14.7%)	107 (15.1%)	0.783
Hypertension, yes, n (%)	385 (16.3%)	266 (16.1%)	119 (16.8%)	0.653
Diabetes, yes, n (%)	192 (8.1%)	122 (7.4%)	70 (9.9%)	0.040
Smoke, yes, n (%)	1,070 (45.3%)	751 (45.4%)	319 (45.1%)	0.895
Alcohol, yes, n (%)	535 (22.6%)	369 (22.3%)	166 (23.4%)	0.536
Clinical characteristic				
Tumor type, yes, n (%)				
Lung cancer	429 (18.1%)	307 (18.5%)	122 (17.2%)	0.450
Gastric cancer	504 (21.3%)	351 (21.2%)	153 (21.6%)	0.822
Esophagus cancer	216 (9.1%)	162 (9.8%)	54 (7.6%)	0.096
Colorectal cancer	610 (25.8%)	429 (25.9%)	181 (25.6%)	0.096
Hepatic-biliary-pancreatic cancer	177 (7.5%)	118 (7.1%)	59 (8.3%)	0.307
Gynecological cancer	176 (7.4%)	125 (7.5%)	51 (7.2%)	0.770
Breast cancer	126 (5.3%)	84 (5.1%)	42 (5.9%)	0.394
Other cancer	126 (5.3%)	83 (5.0%)	43 (6.1%)	0.293
TNM stage, n (%)				0.272
I	205 (8.7%)	140 (8.5%)	65 (9.2%)	
II	492 (20.8%)	360 (21.7%)	132 (18.6%)	
III	637 (26.9%)	433 (26.1%)	204 (28.8%)	
IV	1,030 (43.6%)	723 (43.7%)	307 (43.4%)	
Surgery, yes, n (%)	975 (41.2%)	661 (39.9%)	314 (44.4%)	0.045
Radiotherapy, yes, n (%)	146 (6.2%)	103 (6.2%)	43 (6.1%)	0.892
Chemotherapy, yes, n (%)	1,126 (47.6%)	787 (47.5%)	339 (47.9%)	0.873
Albumin, g/L, mean (SD)	37.35 (5.46)	37.37 (5.46)	37.31 (5.50)	0.796
Globulin, g/L, mean (SD)	29.22 (5.72)	29.16 (5.71)	29.37 (5.75)	0.402
AGR, ratio, mean (SD)	1.34 (0.71)	1.35 (0.82)	1.32 (0.33)	0.368
Neutrophil, 10^9^/L, median (IQR)	4.02 (3.25)	4.02 (3.25)	4.01 (3.30)	0.890
Lymphocyte, 10^9^/L, median (IQR)	1.35 (0.89)	1.34 (0.87)	1.37 (0.92)	0.388
WBC, 10^9^/L, mean (SD)	7.23 (7.99)	7.32 (9.27)	7.01 (3.39)	0.378
Platelet, 10^9^/L, median (IQR)	226.00 (118.75)	225.00 (116.00)	228.00 (128.25)	0.601
KPS, mean (SD)	82.87 (15.44)	82.72 (15.30)	83.23 (15.77)	0.456
MAC, cm, mean (SD)	24.98 (3.72)	24.94 (3.75)	25.06 (3.66)	0.453
HGS, kg, mean (SD)	23.38 (10.36)	23.47 (10.41)	23.19 (10.31)	0.537
PG-SGA, mean (SD)	7.43 (4.09)	7.43 (4.09)	7.42 (4.10)	0.971
EORTC QLQ-C30, mean (SD)	52.63 (11.40)	52.61 (11.55)	52.68 (11.55)	0.892

Data are represented as mean [standard deviation (SD)], median [interquartile range (IQR)], or number (%).

BMI, body mass index; KPS, Karnofsky Performance Status; AGR, albumin–globulin ratio; MAC, mid-arm circumference; HGS, handgrip strength; PG-SGA, patient-generated subjective global assessment; SMI, skeletal muscle index; WBC, white blood cell.

### Characteristics of Clinical Baseline Data

Based on the cutoff value, there are 644 (38.89%) patients with low AGR and 1,012 (61.11%) patients with high AGR in training cohort. While in the validation cohort, there were 292 (41.24%) patients with low AGR and 416 (58.76%) patients with high AGR. Low AGR was associated with advanced age, low BMI, low ALB, high GLB, high neutrophils, high WBC, high platelet, low KPS, low MAC, high PG-SGA, and high EORTC QLQ-C30 (**Table 2**). Correlation analysis showed that BMI and HGS were significantly positively correlated with AGR, while BMI and MAC were weakly positively correlated with AGR. In addition, age, NLR, PLR, and PG-SGA were significantly negatively correlated with AGR, which showed good consistency in the two cohorts (**Supplementary Figures 3A, B**). We also further analyzed the relationship between AGR and tumor stage, and we found that AGR gradually decreased with the progression of tumor stage in both the training and validation cohorts (**Supplementary Figures 1B, C**).

**Table 2:** Characteristic of the study patients with cancer cachexia stratified by AGR.

Characteristic	Training cohort			Validation cohort		
	AGR low n = 644	AGR high n = 1,012	*p*	AGR low n = 292	AGR high n = 416	*p*
Population characteristic						
Gender, male, n (%)	393 (61.0%)	580 (57.3%)	0.135	189 (64.7%)	217 (52.2%)	<0.001
Age, years, mean (SD)	60.13 (12.00)	57.67 (11.57)	<0.001	59.18 (13.06)	57.96 (12.79)	0.024
BMI, kg/m^2^, mean (SD)	20.41 (3.07)	21.18 (3.32)	<0.001	20.50 (3.29)	21.14 (3.27)	0.010
Family history	106 (16.5%)	137 (13.5%)	0.101	45 (15.4%)	62 (14.9%)	0.853
Hypertension, yes, n (%)	111 (17.2%)	155 (15.3%)	0.300	56 (19.2%)	63 (15.1%)	0.158
Diabetes, yes, n (%)	56 (8.7%)	66 (6.5%)	0.099	36 (12.3%)	34 (8.2%)	0.068
Smoke, yes, n (%)	299 (46.4%)	452 (44.7%)	0.482	142 (48.6%)	177 (42.5%)	0.109
Alcohol, yes, n (%)	142 (22.0%)	227 (22.4%)	0.856	80 (27.4%)	86 (20.7%)	0.038
Clinical characteristic						
Tumor type, yes, n (%)			<0.001			<0.001
Lung cancer	143 (22.2%)	163 (16.1%)		69 (23.6%)	54 (13.0%)	
Gastric cancer	116 (18.0%)	233 (23.0%)		49 (16.8%)	106 (25.5%)	
Esophagus cancer	62 (9.6%)	100 (9.9%)		27 (9.2%)	27 (6.5%)	
Colorectal cancer	159 (24.7%)	270 (26.7%)		74 (25.3%)	107 (25.7%)	
Hepatic-biliary-pancreatic cancer	62 (9.6%)	56 (5.5%)		34 (11.6%)	25 (6.0%)	
Gynecological cancer	51 (7.9%)	74 (7.3%)		13 (4.5%)	38 (9.1%)	
Breast cancer	15 (2.3%)	69 (6.8%)		5 (1.7%)	37 (8.9%)	
Other cancer	36 (5.6%)	47 (4.6%)		21 (7.2%)	22 (5.3%)	
TNM stage, n (%)			<0.001			<0.001
I	40 (6.2%)	100 (9.9%)		14 (4.8%)	51 (12.3%)	
II	105 (16.3%)	255 (25.2%)		39 (13.4%)	93 (22.4%)	
III	148 (23.0%)	285 (28.2%)		65 (22.3%)	139 (33.4%)	
IV	351 (54.5%)	372 (36.8%)		174 (59.6%)	133 (32.0%)	
ALB, g/L, mean (SD)	33.77 (4.99)	39.66 (4.41)	<0.001	33.84 (4.83)	39.74 (4.56)	<0.001
GLB, g/L, mean (SD)	33.45 (5.31)	26.42 (4.01)	<0.001	33.81 (5.37)	26.25 (3.55)	<0.001
Neutrophil, 10^9^/L, median (IQR)	4.65 (3.98)	3.58 (2.74)	<0.001	5.19 (4.03)	3.52 (2.38)	<0.001
Lymphocyte, 10^9^/L, median (IQR)	1.30 (0.85)	1.36 (0.88)	0.333	1.35 (0.90)	1.40 (0.90)	0.351
WBC, 10^9^/L, mean (SD)	7.93 (4.19)	6.93 (11.38)	0.033	7.95 (4.09)	6.34 (2.61)	<0.001
Platelet, 10^9^/L, median (IQR)	254.0 (139.5)	211.0 (101.75)	<0.001	253.00 (170.0)	214.00 (112.5)	<0.001
KPS, mean (SD)	79.53 (16.65)	84.74 (14.01)	<0.001	79.28 (17.07)	86.01 (14.17)	<0.001
MAC, cm, mean (SD)	24.56 (3.71)	25.18 (3.77)	0.001	24.63 (3.77)	25.37 (10.60)	<0.001
HGS, kg, mean (SD)	21.81 (9.37)	24.53 (10.89)	<0.001	22.37 (9.83)	23.76 (10.60)	0.078
PG-SGA, mean (SD)	8.28 (4.22)	6.89 (3.92)	<0.001	8.30 (4.30)	6.8 (3.85)	<0.001
EORTC QLQ-C30, mean (SD)	54.69 (12.40)	51.29 (10.77)	<0.001	55.10 (11.83)	50.99 (10.13)	<0.001

Data are represented as mean [standard deviation (SD)], median [interquartile range (IQR)], or number (%). For AGR, low <1.24; high ≥1.24.

BMI, body mass index; AGR, albumin–globulin ratio; ALB, albumin; GLB, globulin; KPS, Karnofsky Performance Status; MAC, mid-arm circumference; HGS, handgrip strength; PG-SGA, patient-generated subjective global assessment.

### Evaluation of the Prognostic Value of Albumin–Globulin Ratio in Patients With Cancer Cachexia

**Figure 3:**
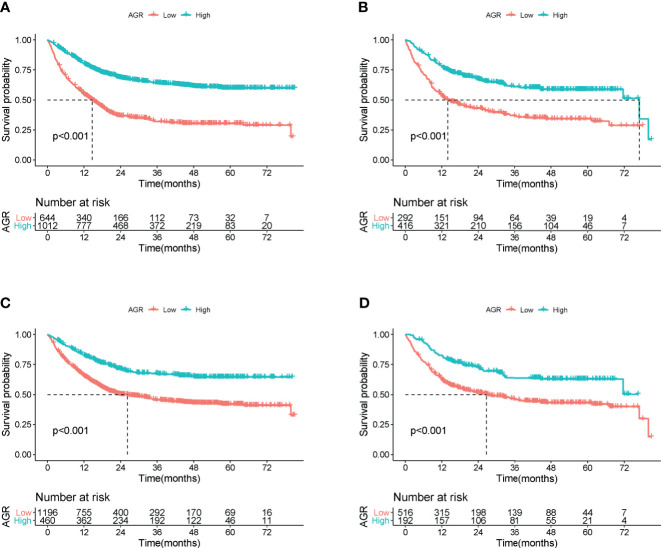
Overall survival of cachexia patients by cutoff of AGR in training and validation cohort. **(A)** AGR cutoff of 1.24 at training cohort. **(B)** AGR cutoff of 1.24 at validation cohort. **(C)** Reference AGR cutoff of 1.5 at training cohort. **(D)** Reference AGR cutoff of 1.5 at validation cohort. AGR, albumin–globulin ratio.

In the training cohort, the median follow-up time was 18.00 (0.03–81.05) months. At the last follow-up, a total of 754 patients (45.53%) died, including 414 patients with low AGR and 340 patients with high AGR. Patients with low AGR had significantly lower OS than those with high AGR (37.71% *vs.* 66.40%, *p* < 0.001) (**Figure 3A**). We also evaluated the reference AGR cutoff value of 1.5. Patients with AGR <1.5 also had significantly lower OS than those with high AGR (48.75% *vs.* 69.35%, *p* < 0.001) (**Figure 3C**). It was worth noting that the survival curve with the AGR cutoff value of 1.24 had a greater degree of opening and closing than those with the reference cutoff value of 1.5. That is to say, the AGR cutoff value of 1.24 was more effective for the prognostic stratification of patients with cancer cachexia. We also compared the survival rates of patients with low and high AGRs in different cancers by Kaplan–Meier survival curve, and the results showed that AGR can effectively stratify the prognosis of patients with various cancers (**Supplementary Figure 4**). In addition, we further performed a stratified analysis based on TNM stage. For I–IV stage patients, the OS of the low AGR group was significantly lower than that of the high AGR group (**Supplementary Figure 5A**). In the univariate analysis, various clinical characteristics were related to the prognosis of patients with cancer cachexia, but multivariate analysis showed that only male, advanced age, advanced TNM stage, low AGR, no surgery, high platelet, low KPS, low HGS, high PG-SGA, high EORTC QLQ-C30 were independent risk factors affecting the prognosis of patients with cancer cachexia (**Supplementary Table 1**, training cohort). Moreover, the trend test showed that AGR was an independent factor affecting the all-cause mortality of patients with cancer cachexia in both classification and continuous methods. Compared with the lowest quartile (Q1: <1.115), quartile 2 (1.115–1.317), quartile 3 (1.317–1.524), and quartile 4 (>1.524) were all negatively associated with poor prognosis (p_trend_ < 0.001) (**Table 3**, training cohort).

**Table 3:** The association between AGR and hazard ratio of cachexia patients.

AGR	Training cohort				Validation cohort			
	Model a		Model b		Model a		Model b	
	HR 95%CI	*p*	HR 95%CI	*p*	HR 95%CI	*p*	HR 95%CI	*p*
As continuous (per SD)	0.536 (0.420, 0.684)	<0.001	0.545 (0.426, 0.697)	<0.001	0.507 (0.361, 0.713)	<0.001	0.528 (0.374, 0.745)	<0.001
By reference AGR cutoff								
Low (~1.50)	Ref		Ref		Ref		Ref	
High (1.50~)	0.591 (0.491, 0.712)	<0.001	0.589 (0.489, 0.710)	<0.001	0.725 (0.549, 0.957)	0.023	0.735 (0.554, 0.975)	0.033
By AGR cutoff								
Low (~1.24)	Ref		Ref		Ref		Ref	
High (1.24~)	0.482 (0.416, 0.559)	<0.001	0.480 (0.414, 0.557)	<0.001	0.688 (0.551, 0.860)	0.001	0.701 (0.559, 0.880)	0.002
Interquartile								
Q1 (~1.115)	Ref		Ref		Ref		Ref	
Q2 (1.115~1.317)	0.699 (0.582, 0.839)	<0.001	0.688 (0.573, 0.827)	<0.001	0.908 (0.686, 1.202)	0.500	0.927 (0.697, 1.232)	0.601
Q3 (1.317~1.524)	0.447 (0.363, 0.549)	<0.001	0.448 (0.364, 0.551)	<0.001	0.663 (0.486, 0.906)	0.010	0.678 (0.494, 0.930)	0.016
Q4 (1.524~)	0.429 (0.346, 0.532)	<0.001	0.418 (0.336, 0.519)	<0.001	0.605 (0.439, 0.835)	0.002	0.622 (0.449, 0.862)	0.004
*p* for trend		<0.001		<0.001		0.005		0.010

Model a: adjusted by gender, age, BMI, and TNM stage. Model b: adjusted by gender, age, BMI, TNM stage, surgery, radiotherapy, chemotherapy, family history, hypertension, diabetes, smoke, and alcohol.

BMI, body mass index.

### Validation of the Prognostic Value of Albumin–Globulin Ratio in Patients With Cancer Cachexia

In the validation cohort, the median follow-up time was 19.2 (0.07–80.79) months. During the follow-up, a total of 336 (47.46%) of patients died, including 180 patients with low AGR and 156 patients with high AGR. Patients with AGR <1.24 had significantly lower OS than those with AGR ≥1.24 (38.36% *vs.* 62.50%, *p* < 0.001) (**Figure 3B**). Similarly, patients with reference AGR <1.5 also had significantly lower OS than those with reference AGR ≥1.5 (47.29% *vs.* 66.67%, p < 0.001) (**Figure 3D**). In the stratified analysis of TNM stage, for III–IV stage patients, OS was significantly lower in the AGR <1.24 group than in the AGR ≥1.24 group, while no significant difference was observed in I–II stage patients (**Supplementary Figure 5B**). Univariate and multivariate survival analyses showed that AGR was an independent factor affecting the prognosis of patients with cancer cachexia (**Supplementary Table 1**, validation cohort). In the trend test, after confounding parameters were adjusted, both the multiple and continuous classifications of AGR were independent factors affecting the prognosis of patients with cancer cachexia. Concordantly, the hazard ratios for all-cause mortality decreased progressively to 0.927 (0.697, 1.232), 0.678 (0.494, 0.930), and 0.622 (0.449, 0.862) when the AGR was divided into quartiles (p_trend_ = 0.010) (**Table 3**, validation cohort).

### Stratified Analysis and Sensitive Analysis

We performed a stratified analysis of potential influencing factors to evaluate the relationship between AGR and all-cause mortality in each subgroup (**Supplementary Table 2**). Overall, the association between low AGR and increased risk of death was consistent in each subgroup of patients with cancer cachexia. In addition, there were interactions between AGR and age, cancer type, and ALB. We further performed the combined survival analysis of AGR and interaction covariates. Whether in training cohort or validation cohort (**Supplementary Figures 7A, B**), the combination of AGR and interaction covariates could still have a good prognostic cumulative effect, which could enhance the prognostic stratification to a certain extent. Our research showed that AGR can be used to predict the prognosis of patients with cancer cachexia, but sensitivity analysis of potential influencing factors was still needed to assess the potential impact on the overall results. First, we intercepted the 6-month short-term prognosis for a sensitivity analysis. The results showed that AGR had a strong predictive ability in predicting short-term outcomes in both training and validation cohorts. Liver and immune diseases also have an impact on AGR, so we also excluded patients with liver and immune disorders for a sensitivity analysis. The results also revealed that AGR was an independent prognostic factor affecting patients with cancer cachexia. Finally, we excluded patients who died within 3 months. It was still observed that the overall risk of death in patients with cancer cachexia gradually decreased as the AGR increased (**Supplementary Tables 3** and **4**).

### Comparison of Albumin–Globulin Ratio and Other Malnutrition Evaluation Tools in Predicting Prognosis

**Figure 4:**
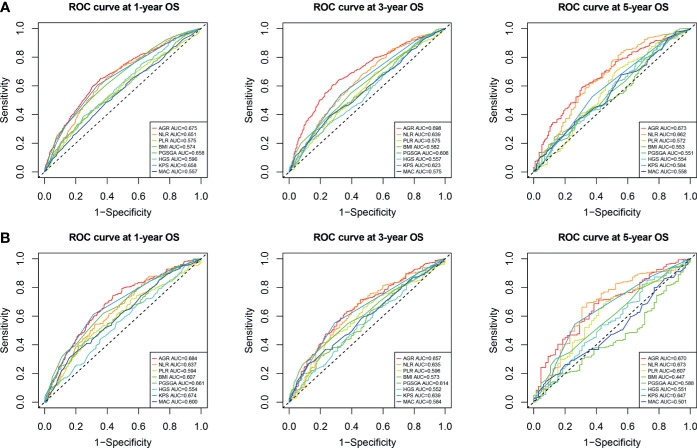
Comparison of the ability of malnutrition evaluation tools in predicting prognosis of cachexia patients using ROC curves. **(A)** ROC curve at 1-, 3-, and 5-year OS points in training cohort. **(B)** ROC curve at 1-, 3-, and 5-year OS points in validation cohort. ROC, receiver operating characteristic; OS, overall survival.

We compared the ability of several serum proteins to predict the prognosis of patients with cancer cachexia through the time-dependent ROC curve. In the training cohort (**Supplementary Figures 6A, B**). The area under the curve (AUC) of AGR (3-year AUC, 0.698; 5-year AUC, 0.673) was higher than that of ALB (3-year AUC, 0.633; 5-year AUC, 0.618), GLB (3-year AUC, 0.577; 5-year AUC, 0.621), and TB (3-year AUC, 0.515; 5-year AUC, 0.519). In the validation cohort (**Supplementary Figures 6C, D**), the AUC of AGR (3-year AUC, 0.657; 5-year AUC, 0.671) was also higher than that of ALB (3-year AUC, 0.638; 5-year AUC, 0.618), GLB (3-year AUC, 0.606; 5-year AUC, 0.602), and TB (3-year AUC, 0.485; 5-year AUC, 0.532). Then, we further compared commonly malnutrition evaluation tools. Compared with other tools, AGR had good prognostic prediction ability, and its prediction performance ranked in the forefront of all prediction tools at 1-, 3-, and 5-year OS points, regardless of whether in the training cohort (**Figure 4A**) or the validation cohort (**Figure 4B**). The effect of malnutrition evaluation tools on all-cause mortality of patients with cancer cachexia is shown in **Tables 4** and **5**. As can be seen from the discriminant index including C-statistic, continuous net reclassification improvement (cNRI), and integrated discrimination improvement (IDI), AGR was better than other evaluation tools at predicting mortality in both training and validation cohorts. For mortality risk prediction, each of the malnutrition evaluation tools provided significant incremental prognostic value for TNM stage. The incremental value of AGR was considerable and statistically significant in both training and validation cohorts (all *p* < 0.05).

**Table 4:** Comparative analysis of the discrimination of each malnutrition score for all-cause mortality in training cohort.

Discrimination ability	C-statistic		cNRI		IDI	
	Difference	*p*-Value	Difference	*p*-Value	Difference	*p*-Value
AGR	Ref		Ref		Ref	
ALB	−0.019 (−0.038, −0.001)	0.043	−0.094 (−0.292, 0.186)	0.408	−0.020 (−0.070, 0.058)	0.350
GLB	−0.061 (−0.075, −0.046)	<0.001	−0.097 (−0.279, 0.255)	0.398	−0.022 (−0.064, 0.063)	0.448
TB	−0.121 (−0.151, −0.091)	<0.001	−0.250 (−0.338, 0.015)	0.070	−0.073 (−0.126, 0.000)	0.060
NLR	−0.017 (−0.043, −0.008)	0.189	−0.258 (−0.345, 0.123)	0.100	−0.068 (−0.117, 0.009)	0.070
PLR	−0.082 (−0.110, −0.051)	<0.001	−0.249 (−0.345, 0.041)	0.100	−0.070 (−0.119, 0.009)	0.090
PG-SGA	−0.030 (−0.057, −0.003)	0.029	−0.154 (−0.299, 0.085)	0.100	−0.050 (−0.114, 0.031)	0.149
HGS	−0.073 (−0.100, −0.046)	<0.001	−0.194 (−0.296, 0.194)	0.159	−0.055 (−0.110, 0.027)	0.149
KPS	−0.021 (−0.045, −0.005)	0.100	−0.221 (−0.330, 0.033)	0.090	−0.047 (−0.107, 0.016)	0.139
MAC	−0.100 (−0.128, −0.072)	<0.001	−0.172 (−0.277, 0.202)	0.149	−0.058 (−0.113, 0.043)	0.129
**Model performance after the addition of malnutrition indexes to the TNM stage for predicting all-cause mortality**						
Model	C-statistic	*p*-Value	cNRI	*p*-Value	IDI	*p*-Value
TNM stage	0.695 (0.679, 0.712)	<0.001	Ref		Ref	
TNM stage + AGR	0.733 (0.715, 0.751)	<0.001	0.367 (0.195, 0.558)	0.010	0.154 (0.085, 0.236)	<0.001
TNM stage + ALB	0.736 (0.718, 0.754)	<0.001	0.202 (0.031, 0.455)	0.020	0.073 (0.001, 0.164)	0.050
TNM stage + GLB	0.712 (0.694, 0.730)	<0.001	0.383 (0.129, 0.535)	0.020	0.082 (0.035, 0.125)	<0.001
TNM stage + TB	0.710 (0.691, 0.729)	<0.001	0.202 (-0.016, 0.353)	0.060	0.073 (0.001, 0.137)	0.050
TNM stage + NLR	0.737 (0.719, 0.755)	<0.001	0.177 (0.004, 0.351)	0.030	0.005 (-0.004, 0.014)	0.209
TNM stage + PLR	0.715 (0.696, 0.734)	<0.001	0.110 (-0.084, 0.302)	0.398	-0.001 (-0.016, 0.017)	0.975
TNM stage + PG-SGA	0.732 (0.714, 0.750)	<0.001	0.023 (-0.164, 0.194)	0.866	0.024 (-0.030, 0.073)	0.328
TNM stage + HGS	0.718 (0.699, 0.737)	<0.001	0.073 (-0.115, 0.250)	0.517	0.032 (-0.013, 0.081)	0.189
TNM stage + KPS	0.732 (0.713, 0.751)	<0.001	0.220 (0.024, 0.401)	0.030	0.078 (0.008, 0.147)	0.030
TNM stage + MAC	0.708 (0.689, 0.727)	<0.001	0.047 (-0.126, 0.226)	0.488	0.033 (0.004, 0.071)	0.030

cNRI, continuous net reclassification improvement; IDI, integrated discrimination improvement; AGR, albumin–globulin ratio; KPS, Karnofsky Performance Status; MAC, mid-arm circumference; HGS, handgrip strength; PG-SGA, patient-generated subjective global assessment; NLR, neutrophil–lymphocyte ratio; PLR, platelet–lymphocyte ratio.

**Table 5:** Comparative analysis of the discrimination of each malnutrition score for all-cause mortality in validation cohort.

Discrimination ability	C-statistic		cNRI		IDI	
	Difference	*p*-Value	Difference	*p*-Value	Difference	*p*-Value
AGR	Ref		Ref		Ref	
ALB	−0.003 (−0.029, 0.023)	0.818	−0.149 (−0.292, −0.003)	0.03	−0.038 (−0.08, 0.000)	0.040
GLB	−0.060 (−0.084, −0.038)	<0.001	−0.153 (−0.314, −0.022)	0.01	−0.054 (−0.091, −0.023)	<0.001
TB	−0.105 (−0.151, −0.058)	<0.001	−0.253 (−0.361, −0.102)	<0.001	−0.100 (−0.147, −0.044)	<0.001
NLR	−0.022 (−0.062, 0.013)	0.252	−0.261 (−0.352, −0.109)	<0.001	−0.092 (−0.141, −0.046)	<0.001
PLR	−0.067 (−0.118, −0.015)	0.011	−0.281 (−0.375, −0.112)	<0.001	−0.094 (−0.142, −0.033)	<0.001
PG-SGA	−0.020 (−0.057, 0.017)	0.296	−0.125 (−0.285, 0.011)	0.080	−0.054 (−0.110, 0.000)	0.050
HGS	−0.095 (−0.140, −0.053)	<0.001	−0.215 (−0.382, −0.11)	<0.001	−0.086 (−0.152, −0.037)	<0.001
KPS	−0.007 (−0.043, 0.027)	0.699	−0.089 (−0.264, 0.068)	0.289	−0.041 (−0.101, 0.029)	0.149
MAC	−0.065 (−0.111, −0.022)	0.004	−0.211 (−0.326, −0.081)	<0.001	−0.095 (−0.151, −0.036)	<0.001
**Model performance after the addition of malnutrition indexes to the TNM stage for predicting all-cause mortality**						
Model	C-statistic	*p*-Value	cNRI	*p*-Value	IDI	*p*-Value
TNM stage	0.720 (0.697, 0.743)	<0.001	Ref		Ref	
TNM stage + AGR	0.750 (0.725, 0.774)	<0.001	0.164 (0.010, 0.287)	0.040	0.027 (0.003, 0.057)	0.010
TNM stage + ALB	0.758 (0.734, 0.783)	<0.001	0.082 (−0.034, 0.215)	0.269	0.019 (−0.001, 0.043)	0.060
TNM stage + GLB	0.730 (0.705, 0.756)	<0.001	0.082 (−0.137, 0.215)	0.259	0.005 (−0.001, 0.018)	0.149
TNM stage + TB	0.737 (0.712, 0.763)	<0.001	−0.048 (−0.173, 0.108)	0.577	−0.001 (−0.009, 0.012)	0.756
TNM stage + NLR	0.757 (0.732, 0.783)	<0.001	0.097 (−0.305, 0.234)	0.458	0.001 (−0.004, 0.007)	0.597
TNM stage + PLR	0.743 (0.718, 0.768)	<0.001	0.158 (0.036, 0.270)	0.020	0.007 (−0.003, 0.023)	0.249
TNM stage + PG-SGA	0.757 (0.731, 0.783)	<0.001	0.061 (−0.127, 0.183)	0.826	0.01 (−0.005, 0.031)	0.239
TNM stage + HGS	0.736 (0.710, 0.762)	<0.001	0.042 (−0.104, 0.170)	0.627	0.005 (−0.006, 0.029)	0.488
TNM stage + KPS	0.758 (0.733, 0.783)	<0.001	0.281 (−0.010, 0.373)	0.060	0.019 (−0.004, 0.044)	0.109
TNM stage + MAC	0.742 (0.717, 0.767)	<0.001	−0.089 (−0.218, 0.090)	0.398	−0.006 (−0.015, 0.009)	0.318

cNRI, continuous net reclassification improvement; IDI, integrated discrimination improvement; AGR, albumin–globulin ratio; KPS, Karnofsky Performance Status; MAC, mid-arm circumference; HGS, handgrip strength; PG-SGA, patient-generated subjective global assessment; NLR, neutrophil–lymphocyte ratio; PLR, platelet–lymphocyte ratio.

## Discussion

Recently, the role of AGR, which reflects inflammation and nutritional status, in malignancy has received more and more attention. Suh et al. (22) conducted a large retrospective study of 26,974 generally healthy adults, showing that low AGR was a short- and long-term risk factor for cancer morbidity and mortality. The meta-analysis of LV et al. (8) also found that low pretreated AGR was associated with poor prognosis of cancers and that AGR should be used as a prognostic indicator during cancer treatment. Moreover, Toiyama et al. (23) suggested that AGR is a new independent predictor of early recurrence in patients with curable gastric cancer. Although AGR has been confirmed to be related to the mortality of many cancers, there is no evidence report on the relationship between AGR and cancer cachexia. In this study, we conducted a large multicenter cohort study for the first time, including 40 clinical centers, involving 2,364 patients with cancer, to explore the prognostic value of AGR in patients with cancer cachexia.

In our study, AGR was suppressed in patients with cancer cachexia, especially in gastrointestinal tumors with a higher incidence of malnutrition, which indicated that the conventional AGR cutoff value may not accurately reflect the AGR level of patients with cancer cachexia. We calculated the specific AGR cutoff value of 1.24 for patients with cachexia cancer. Based on this cutoff value, AGR can better distinguish the poor prognosis of patients with cancer cachexia and still has good value in the prognostic assessment of individual cancer species. Compared with the reference AGR of 1.5, the AGR of 1.24 was more effective in stratifying the prognosis of patients with cancer cachexia. These evidences suggested that AGR of 1.24 is more suitable for patients with cancer cachexia. According to previous studies, the cutoff value of AGR is different for different types of cancers. A meta-analysis in 2018 found that AGR cutoff value in various cancers fluctuated between 0.9 and 1.93, and the vast majority of cutoff value was less than 1.5 (8). The cutoff value of AGR in our study was within the range of these values and at a low level, which to a certain extent indicated that our determined cutoff value was reliable. In addition, we observed an obvious correlation between low AGR and advanced age, low BMI, advanced TNM stage, low ALB, high neutrophils, high platelet, low KPS, low MAC, high PG-SGA, and high EORTC QLQ-C30. TNM stage indicated the malignant degree and tumor burden; advanced age, low BMI, and low ALB indicated poor nutritional status; high neutrophils and high platelets indicated severe cancer-related inflammation; low KPS, low MAC, high PG-SGA, and high EORTC QLQ-C30 reflected the decline in the quality of life. Thus, the low AGR may reflect greater tumor burden, poor nutritional, and advanced inflammatory status, which may be associated with adverse clinical outcomes.

The TNM stage system is currently recognized as the most reliable tool for evaluating the prognosis of patients with malignancies. However, there are reports that patients with the same TNM stage may still have different clinical outcomes, which indicates that under the same TNM stage, other prognostic markers need to be evaluated to achieve a more accurate prognostic assessment (24). In our study, AGR decreased with the progression of TNM stage and could further identify high-risk patients in the advanced cancer subgroup. This may be because advanced patients often have a higher tumor burden and a higher systemic inflammatory. In addition, due to increased consumption and reduced food intake, the nutritional status of advanced patients has also declined. Our study has confirmed that AGR was an independent prognostic factor for patients with cancer cachexia and was an effective prognostic stratification tool in most subgroups. Moreover, we also demonstrated that AGR was an effective predictor of short-term prognosis in patients with cancer cachexia, and the predictive effect of AGR in predicting prognosis in patients with cancer cachexia is not affected by related mixed diseases.

In patients with cancer, high catabolism is accompanied by an increase in overall protein conversion, characterized by accelerated glycogen, protein, and fat decomposition, as well as increased synthesis of immune and inflammatory proteins. Serum ALB level is affected by liver synthesis. When the body has severe inflammation, the liver preferentially synthesizes inflammatory proteins, which changes the priority of protein synthesis, resulting in a lower ALB concentration (12). In this study, the predictive ability of AGR for the prognosis of patients with cancer cachexia is higher than that of ALB, GLB, or TB alone; and the results of subgroup analysis show that AGR can further distinguish the prognosis of normal ALB group. This may be because AGR in patients with cancer reflects the balance of inflammatory protein and ALB. AGR may be a useful indicator that reflects the acceleration of protein conversion, which can more sensitively and accurately reflect the overall protein decomposition/synthesis/transformation metabolism of patients with cancer, thereby improving the accuracy of predicting the prognosis of patients with cancer cachexia. Interestingly, the order of predictive efficacy was roughly AGR>ALB>GLB>TB, which may be because the change of nutritional and inflammatory status in patients with cancer was more sensitive than the change of immune status, while TB cannot effectively reflect the balance of protein conversion, leading to poor prognosis prediction effect. Currently, there are many malnutrition evaluation tools in clinical practice. Good malnutrition evaluation tools can effectively help clinicians to make further treatment plans for patients. We compared the prognostic efficacy of AGR with the commonly malnutrition evaluation tools for patients with cancer cachexia, and the results showed that the prognostic efficacy of AGR was better than that of most prognostic tools.

This study is the first multi-institution large cohort study to explore the relationship between AGR and cancer cachexia. In addition, the patients were randomly divided into two independent cohorts of training and validation to enhance the reliability of the research. In this study, AGR, as a simple, inexpensive, and easily available biomarker, has been proven to be an independent and powerful prognostic indicator for patients with cancer cachexia. However, there are still some limitations that need to be noted. First, this study was essentially a retrospective study, and the performance of possible selection bias, detection bias, and analysis bias might be confused. Second, due to the lack of data on other inflammatory nutrition-related indicators in this study, such as C-reactive protein and Glasgow prognosis score, it was regrettable that AGR cannot be compared with other inflammation nutrition-related indicators for further prognostic ability, which needed to be further explored in future studies. Finally, the results of this study were based on a retrospective cohort study, and further prospective cohort studies were needed to verify this.

## Conclusion

This study determined the AGR cutoff value of 1.24 in patients with cancer cachexia and confirmed that AGR is an independent prognostic factor affecting patients with cancer cachexia, especially those with advanced stage. Compared with other malnutrition evaluation tools, AGR can effectively stratify the prognosis of patients with cancer cachexia.

## Data Availability Statement

The original contributions presented in the study are included in the article/**Supplementary Material**. Further inquiries can be directed to the corresponding author.

## Ethics Statement

The studies involving human participants were reviewed and approved by the institutional review boards of all participating institutions. The patients/participants provided their written informed consent to participate in this study.

## Author Contributions

H-PS and H-LX: study design and guidance, critical revision of the manuscript, and study supervision. H-LX, QZ, and G-TR: data acquisition and analysis, statistical evaluation of the results, and drafting of the manuscript. Y-ZG, C-LH, M-MS, C-HS, XZ, and X-WZ: data acquisition, analysis, and interpretation. X-RL, K-PZ, TL, MY, MT, and H-XX: software application. All authors contributed to the article and approved the submitted version.

## Funding

This work was supported by the National Key Research and Development Program to H-PS (No. 2017YFC1309200).

## Conflict of Interest

The authors declare that the research was conducted in the absence of any commercial or financial relationships that could be construed as a potential conflict of interest.

## Publisher’s Note

All claims expressed in this article are solely those of the authors and do not necessarily represent those of their affiliated organizations, or those of the publisher, the editors and the reviewers. Any product that may be evaluated in this article, or claim that may be made by its manufacturer, is not guaranteed or endorsed by the publisher.

## Supplementary Material

Supplementary Figure 1 AGR in different cancer types stratified by whether patients with cachexia and different TNM stage of patients with cachexia. **(A)**, AGR in different cancer types; A, training cohort, **(B)**, validation cohort; ns p-value >0.05, *p-value < 0.05, **p-value < 0.01, ***p-value < 0.001.

Supplementary Figure 2 Cut-off of AGR in patients with cancer cachexia.

Supplementary Figure 3 Correlation analysis between AGR and other parameters in training cohort **(A)** and validation cohort **(B)**. AGR, Albumin-Globulin ratio; BMI, body mass index; KPS, Karnofsky Performance Status; MAC, mid-arm circumference; HGS, hand grip strength; PG-SGA, patient-generated subjective nutrition assessment.

Supplementary Figure 4 Kaplan-Meier curves of OS for cachexia patients stratified by low- and high-AGR for multiple cancer types in training cohort (including lung cancer, gastric cancer, esophagus cancer, colorectal cancer, hepatic-biliary-pancreatic cancer, gynecological cancer, breast cancer, and other cancer).

Supplementary Figure 5 Stratified analysis of cachexia patients with high AGR and low AGR based on TNM stage. **(A)**, training cohort, **(B)**, validation cohort.

Supplementary Figure 6 Comparison of the ability of serum protein in predicting prognosis of cachexia patients using ROC curves. **(A)**, ROC curve at 3-year OS point in training cohort, **(B)**, ROC curve at 5-year OS point in training cohort, **(C)**, ROC curve at 3-year OS point in validation cohort, **(D)**, ROC curve at 5-year OS point in validation cohort.

Supplementary Figure 7 Combined survival analysis of AGR and covariates interaction. **(A)**, training cohort, **(B)**, validation cohort.

## References

[1] Ferlay J EM Lam F Colombet M Mery L Piñeros M. Global Cancer Observatory: Cancer Today (2020). Lyon: International Agency for Research on Cancer. Available at: https://gco.iarc.fr/today (Accessed 2021 February).

[2] Sung H Ferlay J Siegel RL. Global Cancer Statistics 2020: GLOBOCAN Estimates of Incidence and Mortality Worldwide for 36 Cancers in 185 Countries. CA Cancer J Clin (2021) 71(3):209–49. doi: 10.3322/caac.21660 33538338

[3] Zhang S Sun K Zheng R Zeng H Wang S Chen R. Cancer Incidence and Mortality in China, 2015. J Natl Cancer Center (2020) 1(1):2–11. doi: 10.1016/j.jncc.2020.12.001 PMC11256613 39036787

[4] von Haehling S Anker SD. Cachexia as a Major Underestimated and Unmet Medical Need: Facts and Numbers. J Cachexia Sarcopenia Muscle (2010) 1(1):1–5. doi: 10.1007/s13539-010-0002-6 21475699 PMC3060651

[5] Argilés JM Busquets S Stemmler B López-Soriano FJ. Cancer Cachexia: Understanding the Molecular Basis. Nat Rev Cancer (2014) 14(11):754–62. doi: 10.1038/nrc3829 25291291

[6] Prado CM Antoun S Sawyer MB Baracos VE. Two Faces of Drug Therapy in Cancer: Drug-Related Lean Tissue Loss and its Adverse Consequences to Survival and Toxicity. Curr Opin Clin Nutr Metab Care (2011) 14(3):250–4. doi: 10.1097/MCO.0b013e3283455d45 21415735

[7] von Haehling S Anker SD. Prevalence, Incidence and Clinical Impact of Cachexia: Facts and Numbers-Update 2014. J Cachexia Sarcopenia Muscle (2014) 5(4):261–3. doi: 10.1007/s13539-014-0164-8 PMC4248411 25384990

[8] Lv GY An L Sun XD Hu YL Sun DW. Pretreatment Albumin to Globulin Ratio can Serve as a Prognostic Marker in Human Cancers: A Meta-Analysis. Clinica Chimica Acta; Int J Clin Chem (2018) 476:81–91. doi: 10.1016/j.cca.2017.11.019 29170102

[9] Chung JW Park DJ Chun SY Choi SH Lee JN Kim BS. The Prognostic Role of Preoperative Serum Albumin/Globulin Ratio in Patients With non-Metastatic Renal Cell Carcinoma Undergoing Partial or Radical Nephrectomy. Sci Rep (2020) 10(1):11999. doi: 10.1038/s41598-020-68975-3 32686760 PMC7371633

[10] Brenner D Blaser H Mak TW. Regulation of Tumour Necrosis Factor Signalling: Live or Let Die. Nat Rev Immunol (2015) 15(6):362–74. doi: 10.1038/nri3834 26008591

[11] Balkwill F. Tumour Necrosis Factor and Cancer. Nat Rev Cancer (2009) 9(5):361–71. doi: 10.1038/nrc2628 19343034

[12] Evans DC Corkins MR Malone A. The Use of Visceral Proteins as Nutrition Markers: An ASPEN Position Paper. Paper. Nutrition in Clinical Practice (2021) 36(1):22–8. doi: 10.1002/ncp.10588 33125793

[13] Gupta D Lis CG. Pretreatment Serum Albumin as a Predictor of Cancer Survival: A Systematic Review of the Epidemiological Literature. Nutr J (2010) 9(1):69. doi: 10.1186/1475-2891-9-69 21176210 PMC3019132

[14] Hu WH Eisenstein S Parry L Ramamoorthy S. Preoperative Malnutrition With Mild Hypoalbuminemia Associated With Postoperative Mortality and Morbidity of Colorectal Cancer: A Propensity Score Matching Study. Nutr J (2019) 18(1):33. doi: 10.1186/s12937-019-0458-y 31253199 PMC6598281

[15] Hill LA Bodnar TS Weinberg J Hammond GL. Corticosteroid-Binding Globulin is a Biomarker of Inflammation Onset and Severity in Female Rats. J Endocrinol (2016) 230(2):215–25. doi: 10.1530/joe-16-0047 PMC5338597 27418032

[16] Meyer EJ Nenke MA Rankin W Lewis JG Torpy DJ. Corticosteroid-Binding Globulin: A Review of Basic and Clinical Advances. Hormone Metab Res = Hormon- und Stoffwechselforschung = Hormones metabolisme (2016) 48(6):359–71. doi: 10.1055/s-0042-108071 27214312

[17] Diakos CI Charles KA McMillan DC Clarke SJ. Cancer-Related Inflammation and Treatment Effectiveness. Lancet Oncol (2014) 15(11):e493–503. doi: 10.1016/s1470-2045(14)70263-3 25281468

[18] Mantovani A Allavena P Sica A Balkwill F. Cancer-Related Inflammation. Nature (2008) 454(7203):436–44. doi: 10.1038/nature07205 18650914

[19] Fearon K Strasser F Anker SD Bosaeus I Bruera E Fainsinger RL. Definition and Classification of Cancer Cachexia: An International Consensus. Lancet Oncol (2011) 12(5):489–95. doi: 10.1016/s1470-2045(10)70218-7 21296615

[20] Wen X Wang M Jiang CM Zhang YM. Anthropometric Equation for Estimation of Appendicular Skeletal Muscle Mass in Chinese Adults. Asia Pac J Clin Nutr (2011) 20(4):551–6. doi: 10.6133/apjcn.2011.20.4.08 22094840

[21] Hu X Zhang L Wang H Hao Q Dong B Yang M. Malnutrition-Sarcopenia Syndrome Predicts Mortality in Hospitalized Older Patients. Sci Rep (2017) 7(1):3171. doi: 10.1038/s41598-017-03388-3 28600505 PMC5466644

[22] Suh B Park S Shin DW Yun JM Keam B Yang HK. Low Albumin-to-Globulin Ratio Associated With Cancer Incidence and Mortality in Generally Healthy Adults. Ann Oncol: Off J Eur Soc Med Oncol (2014) 25(11):2260–6. doi: 10.1093/annonc/mdu274 25057172

[23] Toiyama Y Yasuda H Ohi M Yoshiyama S Araki T Tanaka K. Clinical Impact of Preoperative Albumin to Globulin Ratio in Gastric Cancer Patients With Curative Intent. Am J Surg (2017) 213(1):120–6. doi: 10.1016/j.amjsurg.2016.05.012 27814784

[24] Lea D Håland S Hagland HR Søreide K. Accuracy of TNM Staging in Colorectal Cancer: A Review of Current Culprits, the Modern Role of Morphology and Stepping-Stones for Improvements in the Molecular Era. Scandinavian J Gastroenterol (2014) 49(10):1153–63. doi: 10.3109/00365521.2014.950692 25144865

