# Supplementary figures and images for: Evaluation and Validation of the Prognostic Value of Serum Albumin to Globulin Ratio in Patients With Cancer Cachexia: Results From a Large Multicenter Collaboration
Source: Front Oncol. 2021 Sep 10;11:707705. doi: 10.3389/fonc.2021.707705 (PMC8461248; doi:10.3389/fonc.2021.707705)

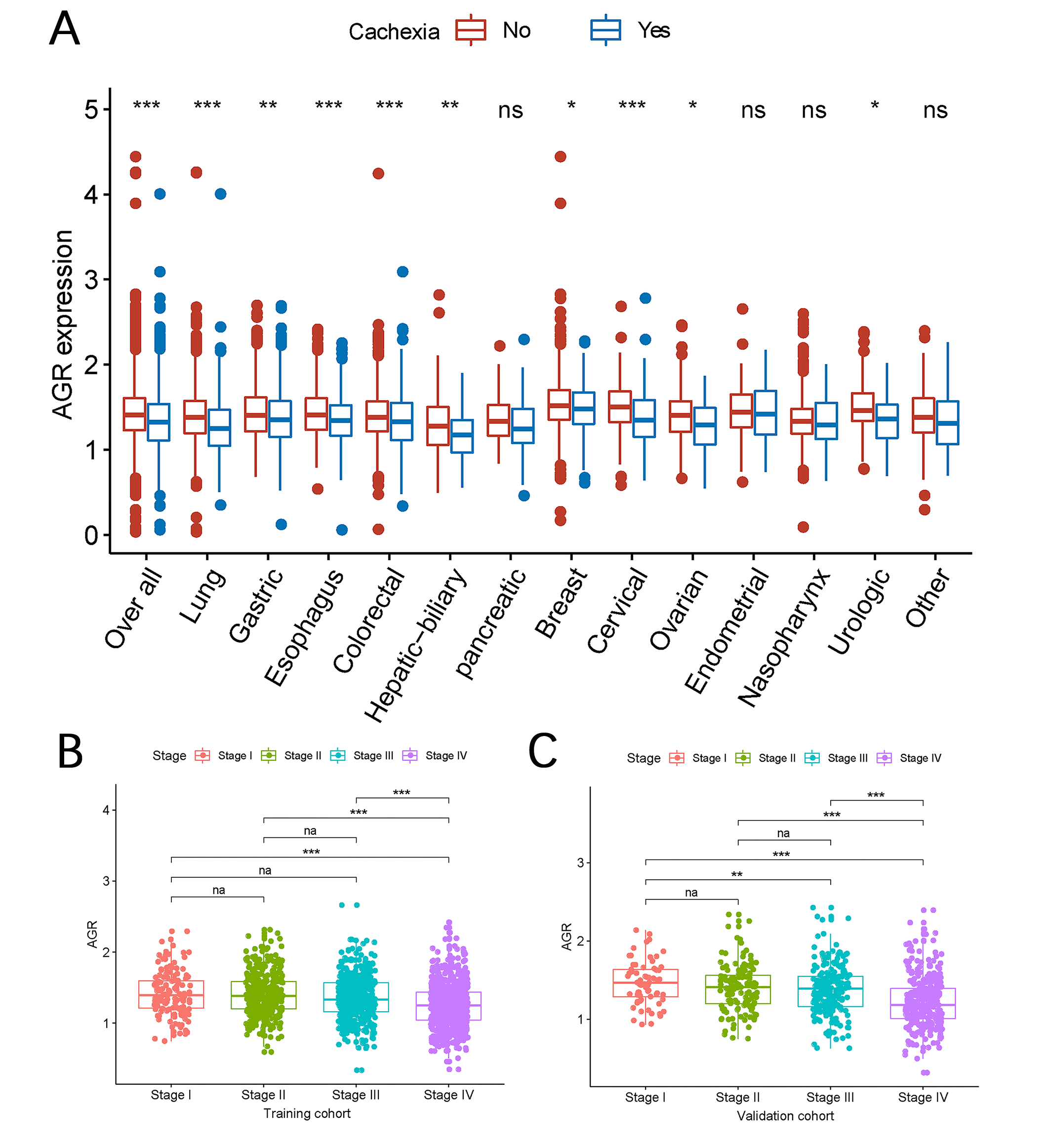

Supplement: Supplementary Figure 1 — AGR in different cancer types stratified by whether patients with cachexia and different TNM stage of patients with cachexia. (A), AGR in different cancer types; A, training cohort, (B), validation cohort; ns p-value >0.05, *p-value < 0.05, **p-value < 0.01, ***p-value < 0.001. [file Image_1.tif]

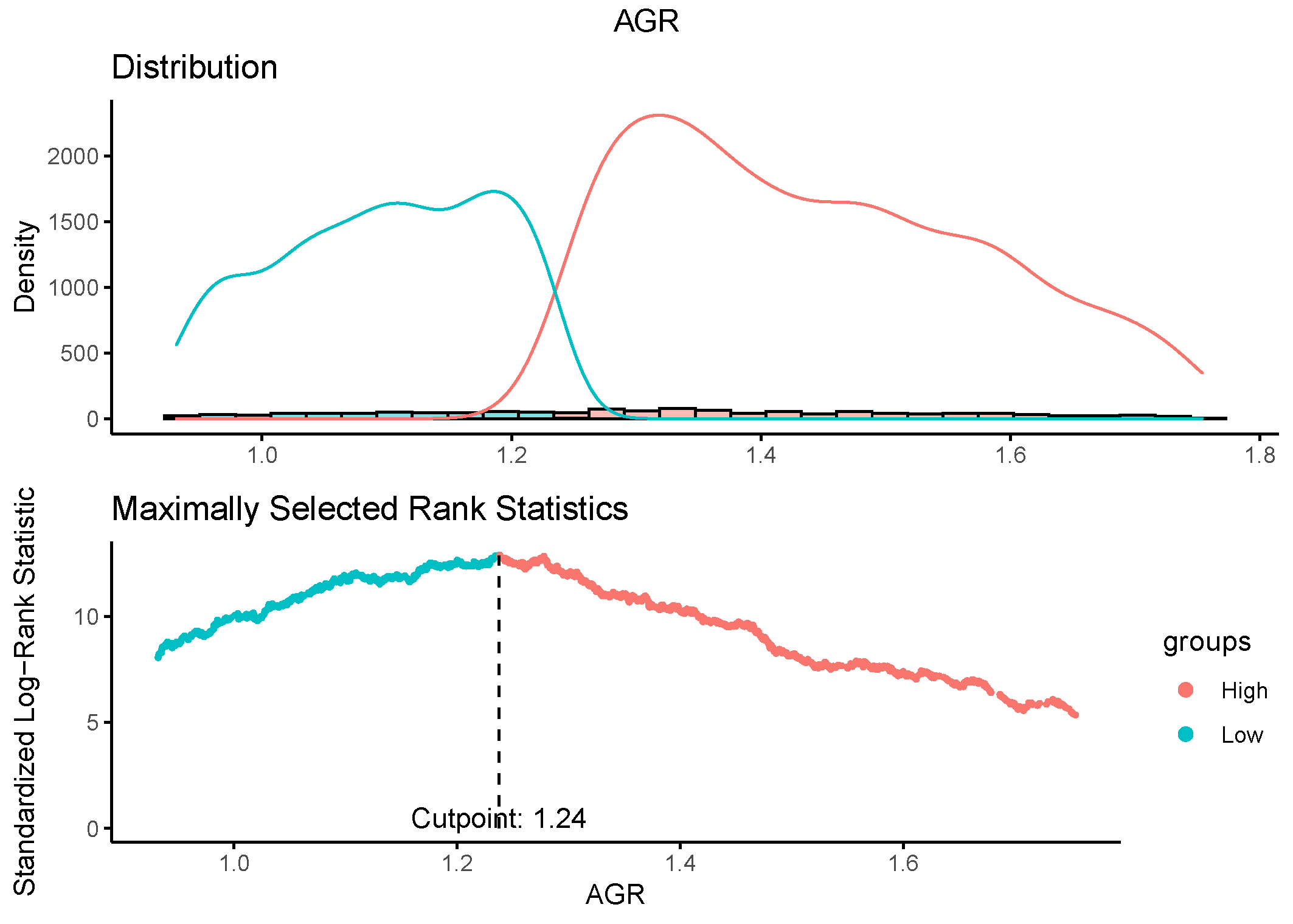

Supplement: Supplementary Figure 2 — Cut-off of AGR in patients with cancer cachexia. [file Image_2.tif]

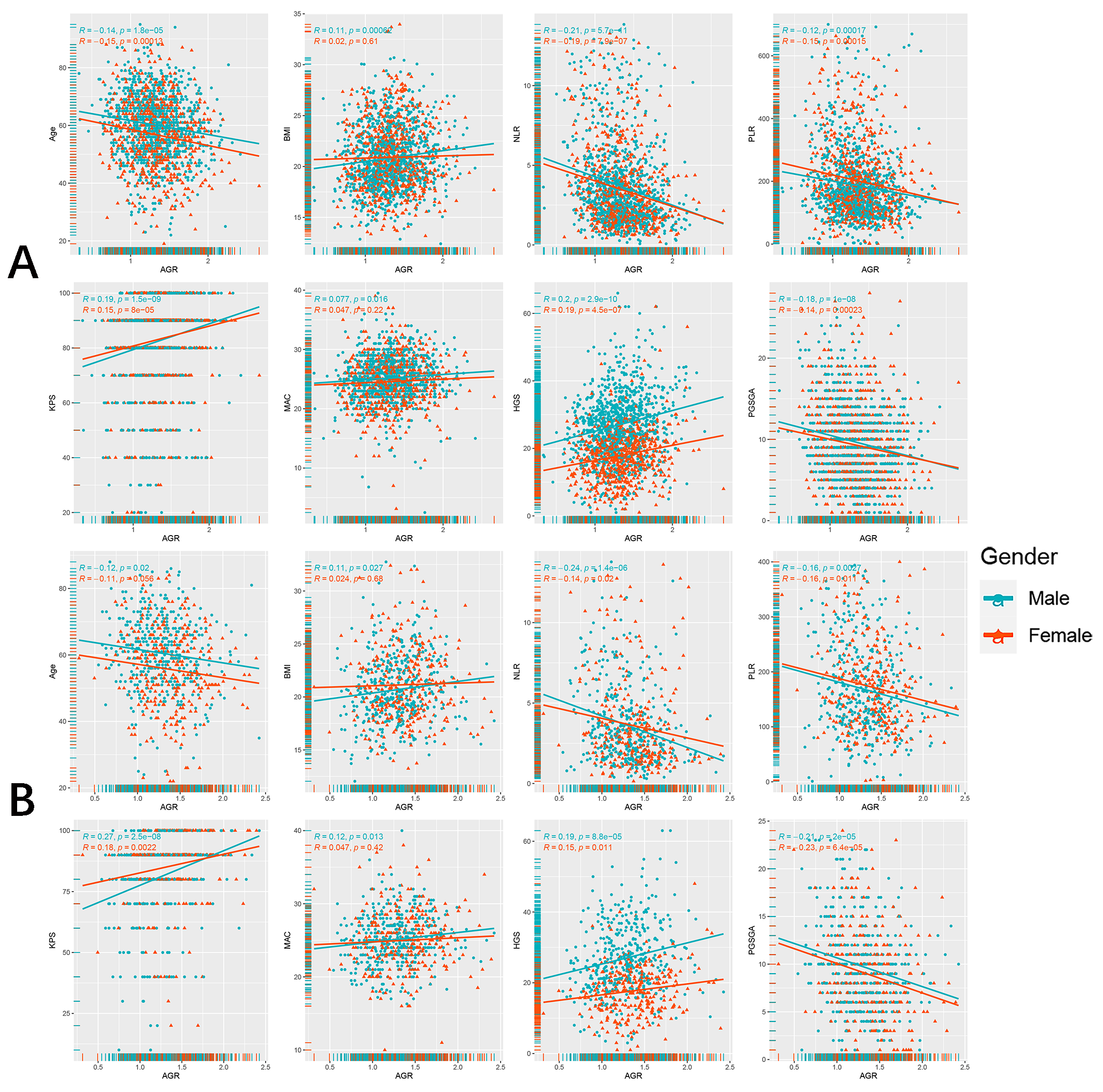

Supplement: Supplementary Figure 3 — Correlation analysis between AGR and other parameters in training cohort (A) and validation cohort (B). AGR, Albumin-Globulin ratio; BMI, body mass index; KPS, Karnofsky Performance Status; MAC, mid-arm circumference; HGS, hand grip strength; PG-SGA, patient-generated subjective nutrition assessment. [file Image_3.tif]

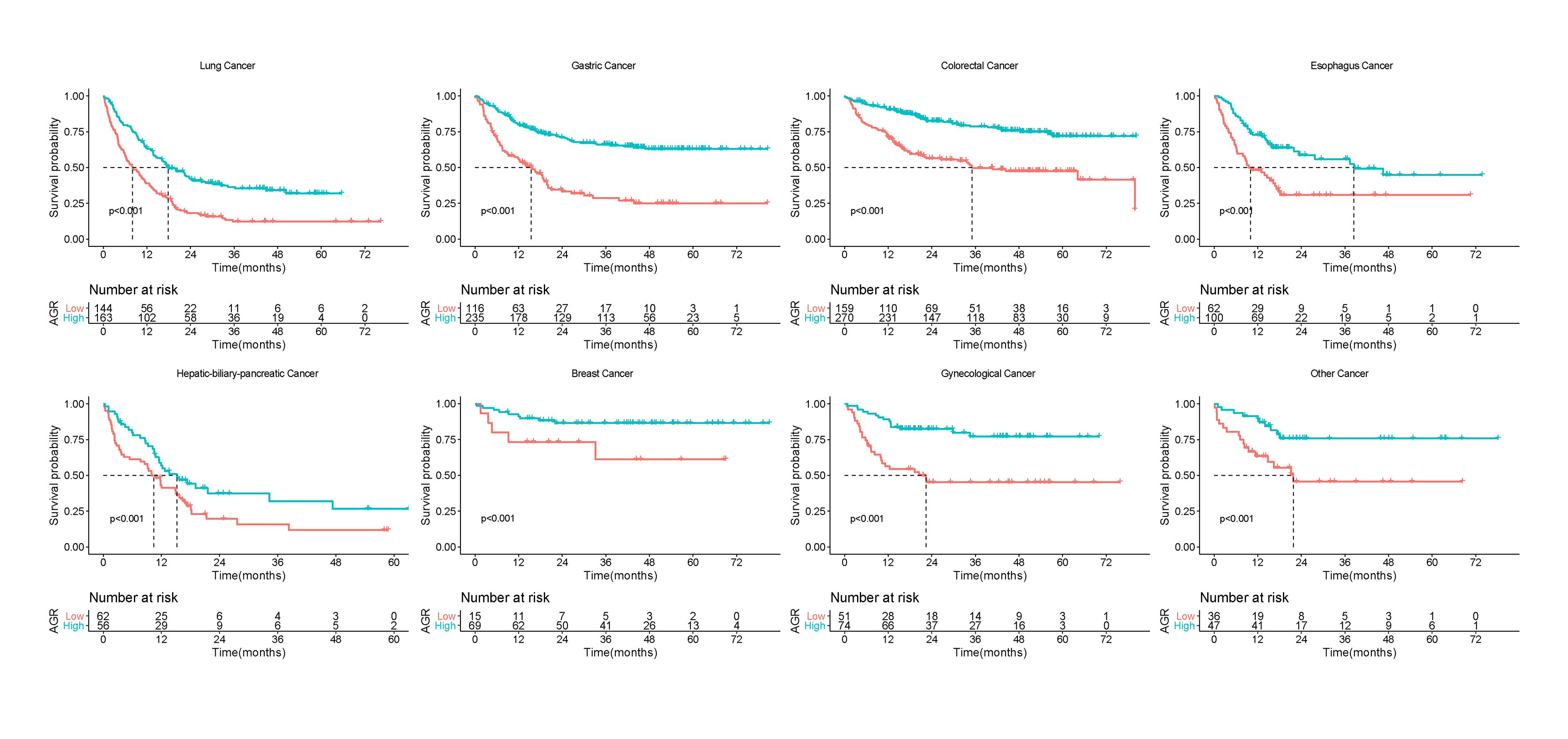

Supplement: Supplementary Figure 4 — Kaplan-Meier curves of OS for cachexia patients stratified by low- and high-AGR for multiple cancer types in training cohort (including lung cancer, gastric cancer, esophagus cancer, colorectal cancer, hepatic-biliary-pancreatic cancer, gynecological cancer, breast cancer, and other cancer). [file Image_4.tif]

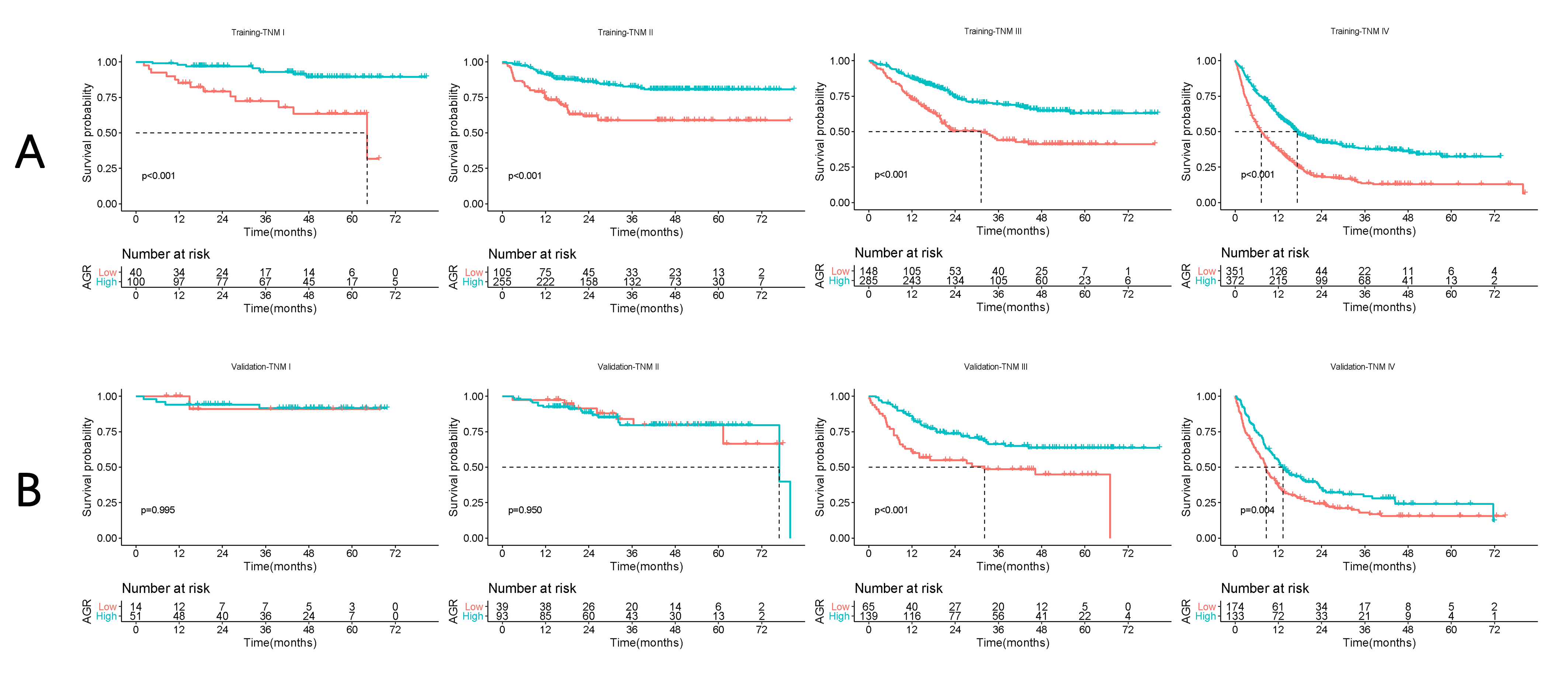

Supplement: Supplementary Figure 5 — Stratified analysis of cachexia patients with high AGR and low AGR based on TNM stage. (A), training cohort, (B), validation cohort. [file Image_5.tif]

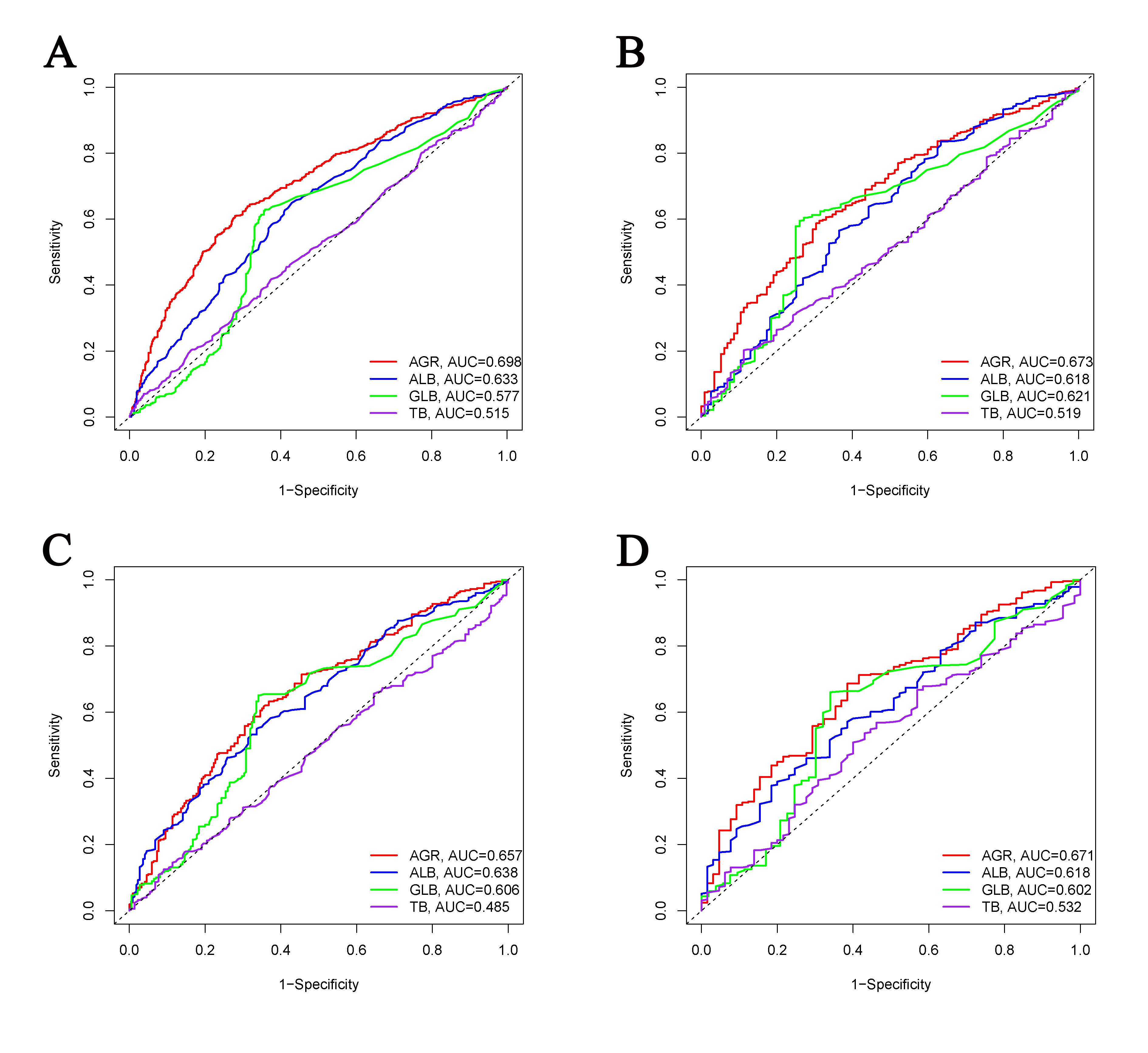

Supplement: Supplementary Figure 6 — Comparison of the ability of serum protein in predicting prognosis of cachexia patients using ROC curves. (A), ROC curve at 3-year OS point in training cohort, (B), ROC curve at 5-year OS point in training cohort, (C), ROC curve at 3-year OS point in validation cohort, (D), ROC curve at 5-year OS point in validation cohort. [file Image_6.tif]

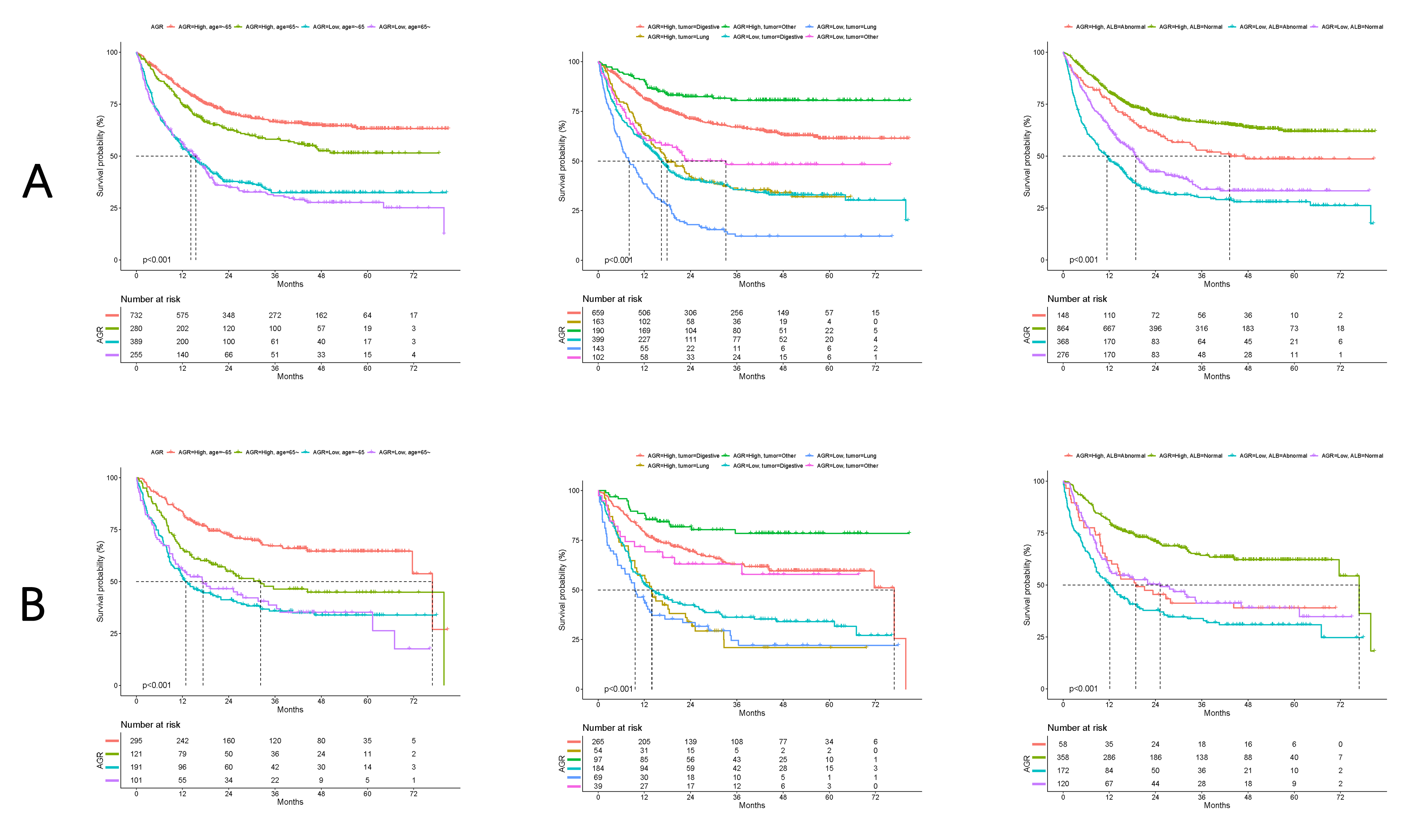

Supplement: Supplementary Figure 7 — Combined survival analysis of AGR and covariates interaction. (A), training cohort, (B), validation cohort. [file Image_7.tif]
