# Supplementary material for: Evaluation and Validation of the Prognostic Value of Serum Albumin to Globulin Ratio in Patients With Cancer Cachexia: Results From a Large Multicenter Collaboration
Source: Front Oncol. 2021 Sep 10;11:707705. doi: 10.3389/fonc.2021.707705 (PMC8461248; doi:10.3389/fonc.2021.707705)
Supplement: Supplementary file 8 [file Table_1.docx]

**Supplementary table 1.** Univariate and multivariate Cox regression analysis of factors associated with overall survival.

| Characteristic | Training cohort | | | | Validation cohort | | | |
| --- | --- | --- | --- | --- | --- | --- | --- | --- |
|  | Univariate analysis | | Multivariate analysis | | Univariate analysis | | Multivariate analysis | |
|  | HR, 95% CI | *p* | HR, 95% CI | *p* | HR, 95% CI | *p* | HR, 95% CI | *p* |
| Gender (Female) | 0.730 (0.629,0.848) | <0.001 | 0.653(0.527,0.810) | <0.001 | 0.548 (0.436,0.690) | <0.001 | 0.637(0.457,0.888) | 0.008 |
| Age (continuous) | 1.019 (1.012,1.025) | <0.001 | 1.011(1.004,1.017) | 0.001 | 1.018 (1.008,1.027) | <0.001 | 1.004(0.994,1.013) | 0.458 |
| BMI (continuous) | 0.933 (0.912,0.955) | <0.001 | 0.980(0.952,1.010) | 0.189 | 0.931 (0.900,0.964) | <0.001 | 0.971(0.926,1.018) | 0.218 |
| Family history | 1.024 (0.838,1.252) | 0.815 |  |  | 1.091 (0.816,1.460) | 0.557 |  |  |
| Hypertension (Yes) | 1.068 (0.883,1.293) | 0.496 |  |  | 1.130 (0.853,1.498) | 0.393 |  |  |
| Diabetes (Yes) | 1.026 (0.783,1.344) | 0.853 |  |  | 1.449 (1.041,2.017) | 0.028 | 1.195(0.845,1.018) | 0.314 |
| Smoke (Yes) | 1.367 (1.185,1.577) | <0.001 | 1.050(0.870,1.266) | 0.613 | 1.406 (1.135,1.743) | 0.002 | 1.071(0.795,1.443) | 0.654 |
| Alcohol (Yes) | 1.153 (0.977,1.362) | 0.092 |  |  | 1.331 (1.045,1.695) | 0.021 | 1.165(0.881,1.541) | 0.283 |
| Tumor stage |  |  |  |  |  |  |  |  |
| I | Ref. |  | Ref. |  | Ref. |  | Ref. |  |
| II | 1.750 (1.083,2.826) | 0.022 | 1.649(1.020,2.667) | 0.041 | 2.398 (0.914,6.295) | 0.076 | 1.914(0.724,5.058) | 0.191 |
| III | 2.972 (1.883,4.689) | <0.001 | 2.619(1.655,4.145) | <0.001 | 6.017 (2.435,14.869) | <0.001 | 4.665(1.874,11.613) | 0.001 |
| IV | 8.885 (5.733,13.770) | <0.001 | 6.586(4.195,10.338) | <0.001 | 18.800 (7.738,45.677) | <0.001 | 12.944(5.248,31.925) | <0.001 |
| Surgery | 0.548 (0.469,0.639) | <0.001 | 0.674 (0.573,0.793) | <0.001 | 0.519 (0.414,0.650) | <0.001 | 0.863 (0.678,1.099) | 0.232 |
| Radiotherapy (Yes) | 1.185 (0.894,1.571) | 0.238 |  |  | 1.138 (0.745,1.739) | 0.549 |  |  |
| Chemotherapy (Yes) | 1.229 (1.065,1.418) | 0.005 | 1.085(0.925,1.274) | 0.315 | 1.187 (0.958,1.470) | 0.118 |  |  |
| AGR (continuous) | 0.320 (0.251,0.408) | <0.001 | 0.710(0.556,0.907) | 0.006 | 0.454 (0.366,0.563) | <0.001 | 0.610(0.421,0.884) | 0.009 |
| Neutrophil (continuous) | 1.003 (0.999,1.006) | 0.073 |  |  | 1.014 (1.001,1.027) | 0.039 | 0.970(0.938,1.003) | 0.077 |
| Lymphocyte (continuous) | 0.934 (0.866,1.008) | 0.078 |  |  | 1.038 (1.015,1.062) | 0.001 | 1.048(1.016,1.081) | 0.003 |
| WBC (continuous) | 1.003 (0.999,1.007) | 0.095 |  |  | 1.078 (1.048,1.110) | <0.001 | 1.036(0.988,1.085) | 0.143 |
| Platelet (continuous) | 1.002 (1.001,1.002) | <0.001 | 1.001(1.000,1.002) | 0.003 | 1.001 (1.000,1.002) | 0.022 | 1.000(0.999,1.001) | 0.558 |
| KPS (continuous) | 0.978 (0.974,0.982) | <0.001 | 0.992(0.986,0.997) | 0.004 | 0.975 (0.969,0.980) | <0.001 | 0.991(0.982,1.000) | 0.062 |
| MAC (continuous) | 0.959 (0.941,0.976) | <0.001 | 1.002(0.977,1.029) | 0.853 | 0.939 (0.912,0.967) | <0.001 | 0.980(0.940,1.023) | 0.354 |
| HGS (continuous) | 0.976 (0.969,0.983) | <0.001 | 0.985(0.976,0.994) | 0.001 | 0.982 (0.971,0.993) | 0.002 | 0.985(0.971,1.000) | 0.048 |
| PG-SGA (continuous) | 1.085 (1.068,1.102) | <0.001 | 1.016(0.997,1.036) | 0.095 | 1.099 (1.071,1.126) | <0.001 | 1.000(0.971,1.030) | 0.982 |
| EORTC QLQ-C30 (continuous) | 1.036 (1.030,1.041) | <0.001 | 1.015(1.007,1.023) | <0.001 | 1.035 (1.026,1.044) | <0.001 | 1.012(0.998,1.027) | 0.096 |

Table note: BMI: body mass index, KPS, Karnofsky Performance Status; AGR, Albumin-Globulin ratio; MAC, mid-arm circumference; HGS, hand grip strength; PG-SGA, patient-generated subjective nutrition assessment.
