# Supplementary material for: Evaluation and Validation of the Prognostic Value of Serum Albumin to Globulin Ratio in Patients With Cancer Cachexia: Results From a Large Multicenter Collaboration
Source: Front Oncol. 2021 Sep 10;11:707705. doi: 10.3389/fonc.2021.707705 (PMC8461248; doi:10.3389/fonc.2021.707705)
Supplement: Supplementary file 9 [file Table_2.docx]

**Supplementary table 2.** The association between AGR (stratified by cut-off points low ~1.24 or high 1.24~) and hazard risk of all-cause mortality in various subgroups.

| Characteristic | Training cohort | | |  | Validation cohort | | |  |
| --- | --- | --- | --- | --- | --- | --- | --- | --- |
|  | no. of patient | *p* | HR, 95%CI | *p* | no. of patient | *p* | HR, 95%CI | *p* |
| Gender |  |  |  | 0.251 |  |  |  | 0.794 |
| Male | 393/580 | <0.001 | 0.514(0.426,0.620) |  | 189/217 | 0.006 | 0.678(0.513,0.895) |  |
| Female | 251/432 | <0.001 | 0.427(0.333,0.548) |  | 103/199 | 0.143 | 0.740(0.495,1.107) |  |
| Age |  |  |  | 0.125 |  |  |  | 0.013 |
| ~65 | 389/732 | <0.001 | 0.429(0.355,0.517) |  | 191/295 | <0.001 | 0.545(0.409,0.725) |  |
| 65~ | 255/280 | <0.001 | 0.548(0.429,0.701) |  | 101/121 | 0.677 | 1.806(0.737,1.599) |  |
| BMI |  |  |  | 0.085 |  |  |  | 0.104 |
| ~18.5 | 185/206 | 0.001 | 0.604(0.453,0.806) |  | 80/87 | 0.008 | 0.521(0.323,0.842) |  |
| 18.5~24 | 365/614 | <0.001 | 0.451(0.370,0.549) |  | 178/252 | 0.020 | 0.710(0.532,0.947) |  |
| 24~ | 94/192 | <0.001 | 0.377(0.249,0.571) |  | 34/77 | 0.563 | 1.260(0.576,2.757) |  |
| Family history |  |  |  | 0.572 |  |  |  | 0.701 |
| No | 538/875 | <0.001 | 0.464(0.395,0.545) |  | 247/354 | 0.011 | 0.721(0.561,0.927) |  |
| Yes | 106/137 | 0.005 | 0.564(0.377,0.842) |  | 45/62 | 0.164 | 0.657(0.363,1.187) |  |
| Diabetes |  |  |  | 0.028 |  |  |  | 0.967 |
| No | 588/946 | <0.001 | 0.503(0.431,0.588) |  | 256/382 | 0.004 | 0.702(0.552,0.891) |  |
| Yes | 56/66 | <0.001 | 0.220(0.120,0.404) |  | 36/34 | 0.604 | 0.824(0.396,1.713) |  |
| hypertension |  |  |  | 0.537 |  |  |  | 0.412 |
| No | 533/857 | <0.001 | 0.487(0.414,0.574) |  | 236/353 | 0.003 | 0.680(0.530,0.873) |  |
| Yes | 111/155 | <0.001 | 0.439(0.303,0.638) |  | 56/63 | 0.287 | 0.731(0.411,1.301) |  |
| Alcohol |  |  |  | 0.330 |  |  |  | 0.287 |
| No | 502/785 | <0.001 | 0.502(0.422,0.596) |  | 212/330 | 0.049 | 0.767(0.588,0.999) |  |
| Yes | 142/227 | <0.001 | 0.404(0.296,0.552) |  | 80/86 | 0.003 | 0.496(0.312,0.789) |  |
| Smoke |  |  |  | 0.981 |  |  |  | 0.598 |
| No | 345/560 | <0.001 | 0.491(0.398,0.607) |  | 150/239 | 0.028 | 0.697(0.505,0.963) |  |
| Yes | 299/452 | <0.001 | 0.467(0.377,0.580) |  | 142/177 | 0.022 | 0.685(0.496,0.946) |  |
| TNM stage |  |  |  | 0.050 |  |  |  | 0.871 |
| I | 40/100 | 0.008 | 0.251(0.090,0.694) |  | 14/51 | 0.639 | 1.929(0.124,29.924) |  |
| II | 105/255 | <0.001 | 0.393(0.250,0.617) |  | 39/93 | 0.946 | 1.036(0.367,2.924) |  |
| III | 148/285 | <0.001 | 0.455(0.329,0.629) |  | 65/139 | 0.006 | 0.516(0.323,0.825) |  |
| IV | 351/372 | <0.001 | 0.520(0.431,0.626) |  | 174/133 | 0.044 | 0.755(0.574,0.993) |  |
| Cancer types |  |  |  | <0.001 |  |  |  | <0.001 |
| Lung | 143/163 | <0.001 | 0.527(0.400,0.694) |  | 69/54 | 0.724 | 1.085(0.689,1.709) |  |
| Digestive | 399/659 | <0.001 | 0.471(0.388,0.572) |  | 184/265 | 0.003 | 0.642(0.480,0.860) |  |
| Other | 102/190 | <0.001 | 0.362(0.224,0.584) |  | 39/97 | 0.541 | 0.791(0.372,1.680) |  |
| Surgery |  |  |  | 0.962 |  |  |  | 0.304 |
| No | 412/583 | <0.001 | 0.498(0.415,0.598) |  | 193/201 | 0.068 | 0.769(0.580,1.019) |  |
| Yes | 232/429 | <0.001 | 0.458(0.351,0.599) |  | 99/215 | 0.012 | 0.607(0.412,0.894) |  |
| Radiotherapy |  |  |  | 0.850 |  |  |  | 0.297 |
| No | 609/944 | <0.001 | 0.479(0.411,0.560) |  | 272/393 | 0.001 | 0.677(0.536,0.855) |  |
| Yes | 35/68 | 0.014 | 0.460(0.248,0.853) |  | 20/23 | 0.088 | 4.143(0.809,21.202) |  |
| Chemotherapy |  |  |  | 0.268 |  |  |  | 0.968 |
| No | 320/549 | <0.001 | 0.479(0.386,0.594) |  | 147/222 | 0.176 | 0.794(0.568,1.109) |  |
| Yes | 324/463 | <0.001 | 0.508(0.412,0.627) |  | 145/194 | 0.011 | 0.664(0.485,0.910) |  |
| Albumin |  |  |  | 0.002 |  |  |  | 0.001 |
| Normal | 368/148 | <0.001 | 0.522(0.426,0.639) |  | 120/358 | 0.012 | 0.666(0.484,0.916) |  |
| Abnormal | 276/864 | <0.001 | 0.565(0.429,0.745) |  | 172/58 | 0.850 | 1.041(0.687,1.577) |  |
| Globulin |  |  |  | 0.089 |  |  |  | 0.077 |
| Normal | 153/759 | <0.001 | 0.405(0.316,0.518) |  | 223/337 | 0.149 | 0.753(0.512,1.107) |  |
| Abnormal | 491/253 | <0.001 | 0.530(0.416,0.676) |  | 79/69 | <0.001 | 0.446(0.284,0.699) |  |

Table note: The model adjusted by gender, age, BMI, TNM stage, surgery, radiotherapy, chemotherapy, family history, hypertension, diabetes, smoke, alcohol, except for the stratifying variable.
