# Supplementary material for: Evaluation and Validation of the Prognostic Value of Serum Albumin to Globulin Ratio in Patients With Cancer Cachexia: Results From a Large Multicenter Collaboration
Source: Front Oncol. 2021 Sep 10;11:707705. doi: 10.3389/fonc.2021.707705 (PMC8461248; doi:10.3389/fonc.2021.707705)
Supplement: Supplementary file 10 [file Table_3.docx]

**Supplementary table 3.** Hazard risk for all-cause mortality in cachexia patients by including short-term patient survival within 3 months, excluding patients dying within 3 months or excluding patients with liver and immune disorders in training cohort.

| Sensitive analysis | Training cohort | | | | | |
| --- | --- | --- | --- | --- | --- | --- |
|  | Short-term patient survival  within 6 months | | Without patients dying within 3 months | | Without liver and immune disorders | |
| AGR | HR 95%CI | *p*-value | HR 95%CI | *p*-value | HR 95%CI | *p*-value |
| As continuous (per SD) | 0.261(0.175,0.391) | <0.001 | 0.713(0.540,0.940) | 0.017 | 0.323(0.250,0.418) | <0.001 |
| By reference AGR cut-off |  |  |  |  |  |  |
| Low (~1.50) | Ref |  | Ref |  | Ref |  |
| High (1.50~) | 0.520(0.374,0.724) | <0.001 | 0.621(0.507,0.761) | <0.001 | 0.579(0.478,0.700) | <0.001 |
| By AGR cut-off |  |  |  |  |  |  |
| Low (~1.24) | Ref |  | Ref |  | Ref |  |
| High (1.24~) | 0.408(0.320,0.522) | <0.001 | 0.512(0.433,0.605) | <0.001 | 0.464(0.398,0.540) | <0.001 |
| Interquartile |  | <0.001 |  | <0.001 |  | <0.001 |
| Q1 (~1.115) | Ref |  | Ref |  | Ref |  |
| Q2 (1.115~1.317) | 0.617(0.464,0.820) | 0.001 | 0.748(0.606,0.923) | 0.007 | 0.681(0.564,0.821) | <0.001 |
| Q3 (1.317~1.524) | 0.417(0.298,0.582) | <0.001 | 0.478(0.378,0.604) | <0.001 | 0.432(0.349,0.534) | <0.001 |
| Q4 (1.524~) | 0.346(0.238,0.504) | <0.001 | 0.460(0.361,0.586) | <0.001 | 0.402(0.322,0.503) | <0.001 |

Table note: Adjusted by gender, age, BMI, TNM stage, surgery, radiotherapy, chemotherapy, family history, hypertension, diabetes, smoke, alcohol.
