# Supplementary material for: Evaluation and Validation of the Prognostic Value of Serum Albumin to Globulin Ratio in Patients With Cancer Cachexia: Results From a Large Multicenter Collaboration
Source: Front Oncol. 2021 Sep 10;11:707705. doi: 10.3389/fonc.2021.707705 (PMC8461248; doi:10.3389/fonc.2021.707705)
Supplement: Supplementary file 11 [file Table_4.docx]

**Supplementary table 4.** Hazard risk for all-cause mortality in cachexia patients by including short-term patient survival within 3 months, excluding patients dying within 3 months or excluding patients with liver and immune disorders in validation cohort.

| Sensitive analysis | Validation cohort | | | | | |
| --- | --- | --- | --- | --- | --- | --- |
|  | Short-term patient survival  within 6 months | | Without patients dying within 3 months | | Without liver and immune disorders | |
| AGR | HR 95%CI | *p*-value | HR 95%CI | *p*-value | HR 95%CI | *p*-value |
| As continuous (per SD) | 0.315(0.171,0.580) | <0.001 | 0.647(0.441,0.948) | 0.026 | 0.553(0.389,0.786) | 0.001 |
| By reference AGR cut-off |  |  |  |  |  |  |
| Low (~1.50) | Ref |  | Ref |  | Ref |  |
| High (1.50~) | 0.424(0.231,0.780) | 0.006 | 0.855(0.633,1.157) | 0.311 | 0.748(0.561,0.996) | 0.047 |
| By AGR cut-off |  |  |  |  |  |  |
| Low (~1.24) | Ref |  | Ref |  | Ref |  |
| High (1.24~) | 0.551(0.379,0.801) | 0.002 | 0.796(0.615,1.030) | 0.083 | 0.717(0.566,0.907) | 0.006 |
| Interquartile |  | 0.003 |  | 0.329 |  | 0.012 |
| Q1 (~1.115) | Ref |  | Ref |  | Ref |  |
| Q2 (1.115~1.317) | 0.923(0.599,1.422) | 0.716 | 0.859(0.611,1.206) | 0.380 | 0.957(0.711,1.288) | 0.772 |
| Q3 (1.317~1.524) | 0.502(0.296,0.851) | 0.010 | 0.798(0.508,1.037) | 0.211 | 0.670(0.482,0.932) | 0.017 |
| Q4 (1.524~) | 0.355(0.184,0.688) | 0.002 | 0.726(0.508,1.037) | 0.078 | 0.634(0.454,0.886) | 0.008 |

Table note: Adjusted by gender, age, BMI, TNM stage, surgery, radiotherapy, chemotherapy, family history, hypertension, diabetes, smoke, alcohol.
